# Inhibition of WNT signaling sensitizes CRC cells to anti-tumor effects of retinoids: basis for new drug combinations in oncology

**DOI:** 10.1080/15384047.2026.2732738

**Published:** 2026-10-08

**Authors:** Victoria O. Hunsu, Caroline O. B. Facey, Lynn M. Opdenaker, Bruce M. Boman

**Affiliations:** a Cawley Center for Translational Cancer Research, Helen F. Graham Cancer Center & Research Institute, Newark, DE, USA; b Department of Biological Sciences, University of Delaware, Newark, DE, USA; c Department of Pharmacology & Experimental Therapeutics, Thomas Jefferson University, Philadelphia, PA, USA

**Keywords:** colorectal cancer, *APC* mutation, drug discovery, WNT signaling, retinoic acid signaling

## Abstract

Our *Goal* is to sensitize human colorectal cancer (CRC) to the differentiation-inducing effects of retinoic acid (RA) agents by suppressing WNT signaling. Previously, we reported that the WNT and RA pathways are functionally linked, and in CRC, *APC* mutation generates a WNT:RA imbalance leading to incomplete differentiation and stem cell (SC) overpopulation. *Hypothesis*: Therapeutically inhibiting WNT signaling enhances the ability of retinoid agents to induce growth suppression and differentiation. We tested drug combinations of: (1) anti-WNT agents (Sulindac & Piroxicam) that produce anti-tumor activity in humans and animals harboring germline *APC* mutations, with (2) RA agents (All-*trans-*RA, 9-*cis* RA) and RA metabolism blocking agent (CYP26A1 inhibitors Liarozole and Talarozole), which inhibit proliferation and induce differentiation of CRC cell lines. *Results:* The combined effect of anti-WNT agents and RA drugs shows additive or synergistic responses in CRC cell lines compared to single agents. Drug combinations decrease the proportion of aldehyde dehydrogenase-positive SCs and increase neuroendocrine cell differentiation in CRC cells that show a synergistic response to treatment. NanoString profiling shows that therapeutically decreasing WNT signaling in CRC cells significantly changes the effect of RA signaling on gene expression. Treating human CRC organoid cultures and PDX mouse models with WNT:RA-based drug combinations led to greater anti-tumor activity as compared to individual agents. *Conclusion*: Inhibiting WNT signaling effectively sensitizes CRC cells to the anti-tumor effects of retinoids. These findings have important clinical significance for designing new drug combinations against CRC.

## Graphical abstract

**Figure:**
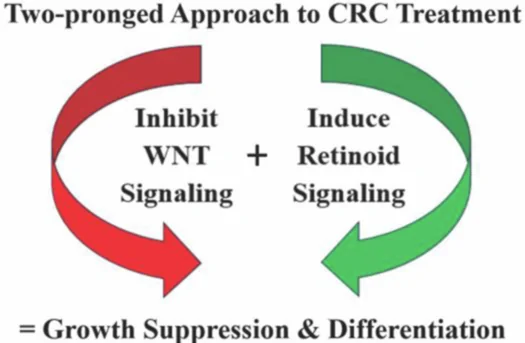

## Introduction

Our objective was to discover how to effectively increase the anti-tumor effects of retinoic acid (RA) signaling in the treatment against colorectal cancer (CRC). This objective arises from the concept of differentiation therapy that aims to change the cancer cell phenotype into a differentiated, non-proliferative phenotype. This concept arose more than 50 y ago^1^ and has maintained momentum over the ensuing years.^2‐8^ More recently, a similar concept, reversion of cancer to a normal tissue state, has emerged.^9^ The hallmark of the success of differentiation therapy has been the treatment of acute promyelocytic leukemia (APL), a hematologic malignancy that is highly curable by retinoic acid (RA) drug combinations.^10‐14^ RA therapy has also been shown to improve the survival of neuroblastoma patients.^15^ ^,^ ^16^ Still, retinoids have not yet been translated into effective systemic treatments for solid tumors. For example, RA-based agents have been evaluated for clinical anti-tumor activity in breast, colorectal, and lung cancers, but with limited success.^17‐22^ Nonetheless, laboratory experiments continue to show that RA agents can trigger differentiation in a variety of cancer cell types, suggesting that they may still be clinically useful. Thus, it is important to explore how solid tumor types might be sensitized to RA’s anti-tumor activity.

Accordingly, we began exploring how CRC cells could be targeted in order to sensitize them to RA’s anti-tumor activity. In this quest, we discovered that a functional link exists between WNT and RA pathways, and, in CRC, adenomatous polyposis coli (*APC*) mutation generates a WNT:RA imbalance that leads to stem cell (SC) overpopulation and incomplete differentiation.^23^ In this situation, *APC* mutation, which occurs in most (~90%) sporadic CRCs, constitutively activates WNT signaling, and an increase in WNT inhibits neuroendocrine cell (NEC) differentiation. Additionally, WNT signaling is activated by *CTNNB1* mutations, which occur in a small number of CRCs (5%–10%). The resulting tumor phenotype is an overpopulation of aldehyde dehydrogenase-positive (ALDH+) SCs and incomplete differentiation of NECs.^24^ Unfortunately, while all-trans RA (ATRA) can typically induce differentiation of ALDH + SCs along the NEC lineage, *APC* mutations inhibit the retinoid-induced NEC differentiation. But, we observed that inducing *wild-type APC* expression in *APC*-mutant CRC cells restores the ability of RA to induce NEC differentiation.^23^

In studying the RA pathway, we have been investigating the idea that dysregulated RA-signaling contributes to the stem cell (SC) overpopulation that drives CRC development. Indeed, we previously reported that ALDH is a marker for normal and malignant human colonic stem cells (SC) and can track SC overpopulation during colon tumorigenesis.^24^ Moreover, we found that ALDH and other RA pathway components are selectively expressed in ALDH + colonic SCs, which indicates that RA signaling mainly occurs in ALDH + SCs.^25^ Indeed, in RA signaling, ATRA is produced by ALDH, which catalyzes the oxidization of vitamin A (retinal or retinaldehyde) into ATRA. Administering ATRA enhances RA signaling to promote the expression of RA-target genes.^26‐28^ In the activation of signaling, RA binds to a receptor complex involving the retinoic acid receptor (RAR) and the retinoid X receptor (RXR). This heterodimer complex then binds to retinoic acid response elements (RAREs) in the promoter regions of RA-target genes.^29‐32^ We previously reported that ATRA-treated CRC cells have reduced proliferation and increased SC differentiation along the neuroendocrine cell (NEC) lineage.^25^ Given that RA signaling plays a key role in regulating colonic SC function, we surmise that discovering novel ways to enhance RA signaling may promote strong anti-tumor activity in CRC cells.

In the WNT pathway, the APC protein is a component of the *β*-catenin destruction complex and is required for down-regulation of WNT signaling [reviewed in 33]. When WNT signaling is inactivated, *β*-catenin is degraded by its multi-protein destruction complex. When WNT signaling is activated, the destruction complex disassembles and *β*-catenin is transported into the nucleus to promote the expression of WNT-target genes. In the activation of signaling, *β*-catenin binds to the transcriptional regulatory complex involving TCF/LEF binding sites, termed WNT response elements (WREs) in the promoter regions of WNT-target genes. Thus, when the APC protein is mutated, the formation of the *β*-catenin destruction complex does not occur, and WNT signaling is constitutively activated. This mechanism explains how constitutive activation of WNT by mutant APC leads to an imbalance in WNT:RA signaling that decreases RA-induced differentiation and increases ALDH + SCs.

A great deal of effort by startups and big Pharma has been invested to develop new agents that target WNT signaling, but most, if not all, of them have proven too toxic to be used in the clinic.^33^ However, some currently existing drugs have been shown to have anti-WNT activity.^34^ In our study, we tested the anti-WNT agents Sulindac^35‐46^ and Piroxicam^47‐51^ because they have been reported to down-regulate WNT signaling and have demonstrated *in vivo* anti-tumor activity. Sulindac and Piroxicam are non-steroidal anti-inflammatory drugs (NSAIDs) that possess cyclooxygenase 2 (COX-2) inhibitory activity. Their antitumor activity has been attributed to COX-2 inhibition, but they also have anti-tumor activity based on inhibition of WNT that is independent of COX-2 inhibition. Table S1 lists the known anti-neoplastic mechanisms for Sulindac and Piroxicam that are independent of COX2. Indeed, a complex interplay exists between the COX2 and WNT signaling pathways.^45^ In the current study, we tested these drugs based on their anti-WNT activity. The chief rationale for this study was based on our previous studies showing that down-regulation of WNT signaling via induction of *wt-APC* in *APC*-mutant CRC cells sensitizes them to the growth-suppressive and differentiation-inducing effects of ATRA.^23^

Hence, we hypothesize that therapeutically inhibiting WNT signaling enhances the ability of retinoid agents to induce growth suppression and differentiation. Thus, our study takes a differentiation therapy approach in which suppressing a cancer driver mechanism restores the original ability of malignant cells to undergo differentiation and growth suppression.^1‐9^ Herein, we took a multi-pronged approach: (1) Test the ability of anti-WNT agents combined with RA agents to inhibit proliferation and induce differentiation of CRC cell lines; (2) Determine if drug combinations have additive or synergistic responses compared to single agents; (3) Evaluate if drug combinations decrease the proportion of ALDH + SCs and increase NEC differentiation; (4) Use NanoString profiling to quantify the effect of anti-WNT and RA agents as well as drug combinations on mRNA expression patterns; and (5) Examine if treatment of human CRC organoid cultures and PDX mouse models with drug combinations shows greater anti-tumor activity as compared to individual agents.

## Materials and methods

Several independent experimental approaches were conducted to determine if the inhibition of WNT signaling sensitizes CRC cells to the anti-tumor effects of retinoids.

### Test agents

Drug sources are as follows: Sulindac, Piroxicam, and Talarozole (Sigma-Aldrich, St Louis, MO), 9-cis retinoic acid (Santa Cruz Biotechnology, Dallas, TX), Liarozole (R&D Chemical, Gonzales, LA), and all-trans retinoic acid (Stem Cell Technologies, Vancouver, BC). Drug doses are listed in each Figure.

### Tissue culture

HT29 (cat # HTB-38), HCT116 (cat # CCL-247), and SW480 (cat # CCL-228) colon cancer cell lines (ATCC, Manassas, VA, USA) were grown in McCoy’s 5 A or Leibovitz 15 medium (ThermoFisher Scientific, Waltham, MA, USA), both supplemented with 10% FBS (Atlanta Biologicals/R&D Systems, Flower Branch, GA, USA) and 1% penicillin/streptomycin (Gibco, Waltham, MA, USA). Upon receipt of cell lines from ATCC, a stock of low-passage vials was prepared and stored (−80 °C) to ensure authenticity throughout the research project. The cells were routinely tested for mycoplasma (Universal mycoplasma detection kit, ATCC Manassas, VA, cat# 30-1012K). The cell cultures were maintained in an incubator at 37 °C with 5% CO_2_.

### Cell proliferation using trypan blue exclusion

Colonic cells were seeded into 12-well plates at 200,000 cells/well for HT29, 200,000 cells/well for SW480, and 80,000 cells/well for HCT116. The cells were incubated in serum-containing medium and allowed to adhere for 24 h. They were then serum-starved for 24 h to bring the cells to the same phase of growth arrest. After serum starvation, the seeded cells were treated with different agents at various drug concentrations, and DMSO (0.1%) was used as a vehicle control. The IC50 (half-maximal inhibitory concentration) doses of the anti-WNT agents and the retinoid agents were determined based on our dose–response experiments.^19^ ^,^ ^20^ ^,^ ^23^ After the predetermined time points, the cells were washed using PBS and collected using Trypsin-EDTA (Fisher Scientific). For each sample, 10 µL of suspended cell culture was mixed with 10 µL of trypan blue (Invitrogen, Waltham, MA, USA). The cell viability was determined using the Trypan blue exclusion method and enumerated using an automated Countess II cell counter (Invitrogen, Waltham, MA, USA). Proliferation analyses were done based on live cell counts. The proliferation index was determined by normalizing treated cells to untreated cells. Biological replicates were performed in triplicate, with three technical replicates per condition. The average was plotted with the standard error of the mean (SEM).

### Determining synergistic, additive, or antagonistic effects of agents

We investigated the antiproliferative effects of the agents on HT29, HCT116, and SW480 cells. Experimental dosage combinations were derived based on the IC50 doses for each of the drugs. For synergy analyses, we used a range of concentrations that centered around each drug's individual potency, specifically around its IC50. The synergistic, additive, and antagonistic responses were determined based on the Bliss Independence Model.^52^ The effects were calculated as the ratio of the observed response (observed response for the combination of drugs A and B) divided by the predicted response (responses of drug A multiplied by drug B).

### ALDEFLOUR assay for ALDH + stem cells

HT29 and SW480 cells were seeded into 10 cm dishes at 3 million cells/dish, and HCT116 at 1 million cells/dish. After the process of serum starvation and treatments, the protocol was followed according to the manufacturer’s instructions (STEMCELL Technologies).^24^ ^,^ ^25^ ^,^ ^53^ Cells were detached from the culture dish with 0.25% Trypsin-EDTA (Fisher Scientific). The cells were spun for 5 min to pellet, resuspended in 1 ml ALDEFLUOR assay buffer, and counted. One million cells/ml were resuspended in ALDEFLUOR assay buffer to obtain a total of 1 ml of solution. Two tubes were labeled as control and sample. To the control tube, 5 µl of the DEAB inhibitor was added. To both tubes containing cells, 5 µl of activated ALDEFLUOR reagent was added, and immediately, 500 µl of the suspension was taken out and placed in the control tube with the inhibitor. The cells were incubated for 30 min at 37 °C in the dark. After incubation, the cells were spun for 5 min to pellet and washed once with ALDEFLUOR buffer. The cells were resuspended in 500  µl ALDEFLUOR buffer and passed through a BD round-bottom tube with a 50 µm cell strainer. The samples were placed on ice and covered from light until they were ready for analysis on the BD LSRFortessa Flow Cytometer.

### Western blotting and densitometry for evaluating protein expression levels

Treated cells in 10 cm culture dishes were detached using trypsin, spun down, and washed with PBS before being lysed in RIPA buffer (Sigma, St. Louis, MO, USA) mixed with 1 × HALT protease inhibitor cocktail (Thermo Fisher Scientific, Waltham, MA, USA). The total protein concentrations of the cell lysate samples were determined using the Pierce BCA Protein Assay kit (Thermo Fisher Scientific, Waltham, MA, USA). For each sample, cell lysates containing 40  µg of protein were loaded to 4%–20% SDS‒PAGE gel (Bio-Rad, Hercules, CA, USA) for electrophoresis at 200 volts for approximately 40–45 min, followed by transferring at 100 volts for 75–80 min to a 0.45 μm PVDF film (Thermo Fisher Scientific, Waltham, MA, USA). Blots were blocked in BLOTTO solution freshly prepared with 5% nonfat dry milk powder in 1 × Tris-buffered saline (Bio-Rad, Hercules, CA, USA) with 0.1% Tween 20 (Thermo Fisher Scientific, Waltham, MA, USA) for 1  h, before incubation in primary antibodies diluted in 5% BLOTTO on a rocking platform at 4 °C overnight. Solutions of secondary antibodies conjugated to horseradish peroxidase were also prepared in BLOTTO and incubated at room temperature for 1 h on a rocking platform. See Table S2 for the sources and titers of the primary and secondary antibodies. The blots were washed with TBS-T after each antibody incubation. Protein bands were detected using Super Signal West Dura Extended Duration Substrate (Thermo Fisher Scientific, Waltham, MA, USA), followed by image development and densitometry using the Li-Cor Odyssey imager (LI-COR Biosciences, Lincoln, NE, USA) and Image Studio software (LI-COR Biosciences, Lincoln, NE, USA).

### TCF/LEF reporter assay (TOPflash) and dual luciferase assay for WNT-signaling activity

WNT/β-catenin signaling activity was measured using the TCF/LEF reporter lentivirus (BPS Bioscience, San Diego, CA) in HT29, SW480, and HCT116 cells following the manufacturer’s instructions. The cells were seeded into flat white clear-bottom 96-well plates at 18,000 cells/well for HT29, 17,000 cells/well for SW480, and 10,000 cells/well for HCT116. The cells were allowed to adhere for 24 h and then were serum-starved for 24 h. The cells were treated for 48 h, and the firefly and Renilla luciferase assays were performed using the Dual-Glo Luciferase Assay System (Promega, Madison, WI, USA). The luminescence was measured using Tecan NanoQuant Infinite F200 PRO (Tecan Trading AG, Switzerland). The luciferase activity was measured by calculating the ratio of firefly to Renilla luminescence signal. Biological replicates were conducted in triplicate, and the averages were plotted along with the standard error of the mean (SEM) in Microsoft Excel 365 and GraphPad Prism.

### NanoString profiling assay for measuring mRNA expression levels

Cells from the corresponding treatments were washed with PBS and harvested using trypsin, washed with PBS before spinning down, and resuspended in TRIzol LS reagent (Thermo Fisher Scientific, Waltham, MA, USA). Once in TRIzol, the cells were pipetted up and down and incubated at room temperature for 10 min before being transferred to the freezer at −80 °C. The samples were sent to the Wistar Institute Genomics Core Facility for NanoString profiling (NanoString Technologies, Seattle, WA, USA). The “pan-cancer NanoString panel plus” that contains 800 targets plus an additional 55 custom set of genes, was chosen for our profiling. The NanoString data was analyzed using nSolver 4.0 software (NanoString Technologies, Seattle, WA, USA).

### Organoid culture

Colon cancer organoids were purchased from American Type Culture Collection (ATCC, Manassas, VA). The ATCC line (HCM-CSHL-0257-C18) has a truncation-causing *APC* mutation (chr5:g.112828889C>T; https://hcmi-searchable-catalog.nci.nih.gov/model/HCM-CSHL-0257-C18) and lacks any RA receptor mutations. Further bioinformatics data is available at https://genomes.atcc.org/cell-bio/135fff90666e400e and the HCMI Catalog. Organoids were cultured in diluted Intesticult media according to Stem Cell Technologies' instructions. Organoid cultures were grown in media for 4  d before treatment. On the 4th day of culture, the organoids were treated with the test agents for 72 h. The treatment concentrations are as follows: 9-cis-RA (60 µM) to induce RA signaling; Sulindac (430 µM), a WNT inhibitor; Liarozole (90 µM), a CYP26A1 inhibitor. The cell viability analysis was done using CYTO 3D Live/Dead Assay Kit (The Well Bioscience, Monmouth Junction, NJ). Images were taken using a Zeiss microscope and analyzed using Zen software.

### Animal studies using PDX mice

We examined drug effectiveness using a *CTNNB1*-mutant human CRC xenograft line (CTG 2359B) grown in mice (see Supplementary Materials for ARRIVE Essential 10 and more details on PDX mice). The CRC xenograft line procurement – CTG-2359 was established by Champions Oncology (Hackensack, NJ). Champions Oncology obtains consented patient samples to engraft, develop, and characterize PDX models from a range of tumor types.^54^ The anti-tumor effect of drugs administered as single agents or in combination was evaluated by assessing toxicity and tumor growth inhibition. Champions Oncology PDX models are fully genotyped and well-characterized in terms of PDX response to single agents. A suitable PDX line was selected for testing at Champions Oncology based on the genotype of the PDX CRC models. Drug responses using PDX mice were evaluated at Champions Oncology. Ethical statement- approval body/number: Champions Oncology IACUC/Implantation of Tumorgrafts and Evaluation of Standard of Care Agents/Proprietary Test Articles in Tumorgraft Models for Translational Oncology Services (IACUC Protocol Title)/2023-TOS-001(IACUC Protocol Number). Champions Oncology uses the gradual displacement method of 30%–70% CO_2_ euthanasia. Eight immunocompromised mice (*nu/nu* Nude) from each treated PDX cohort were compared to 8 PDX control mice. The animals were weighed, and tumor dimensions were measured twice weekly. Champions Oncology formulated oral preparations of drugs. The starting doses for the study agents were: Sulindac 171 mg/kg/day, 9-cis retinoic acid at 3 different doses (5, 10, and 30 mg/kg/d), and Liarozole 40  ppm in a base diet (4 IU/g of vitamin A).

The study endpoint was when the mean tumor volume of the control group (uncensored) reached 1500 mm^3^. If this occurred before Day 28, treatment groups and individual mice were dosed, and measured up to Day 28. If the mean tumor volume of the control group (uncensored) did not reach 1500 mm^3^ by Day 28, then the endpoint for all animals was the day when the mean tumor volume of the control group (uncensored) reached 1500 mm^3^ up to a maximum of Day 60. Outcome measures*:* (1) Tumor growth inhibition (%TGI – see below) of PDXs in response to single drugs (Sulindac, Liarozole, 9-cis retinoic acid) versus placebo. (2) An increase in the anti-CRC effect of a multi-drug combination, compared to giving the drug alone, indicates synergistic or additive activity.

### Statistical analysis

The drug response for each PDX line was calculated based on the %TGI using the difference between the final and initial tumor volumes of the vehicle control as the baseline. The formula used was %TGI = [1-(Treatment volume-final - Treatment volume-initial/Control volume-final—Control volume-initial)] *100. In selecting the specific models to test, the power level calculation for the level of significance (alpha level or the false positive rate) was set at 0.05, where power is calculated based on the probability of identifying an actual difference if one exists (based on false negative rates). Statistics were calculated in Microsoft Excel 365 using the *t*-test function or in GraphPad Prism 10 using one-way ANOVA, two-way ANOVA, or multiple unpaired *t*-tests. All experiments were done at a minimum in triplicate, and statistical significance was based on a *p-*value < 0.05.

## Results

### Anti-WNT agents sensitize CRC cells to the anti-proliferative effects of RA agents

To determine the effects of retinoids and RA metabolism-blocking agents on proliferation, we evaluated them as single agents or in combination with anti-WNT agents (Sulindac and Piroxicam). Screening was done on over 10,000 dose responses of the various agents on HT29, SW480, and HCT116 cells. All three CRC cell lines have mutations that activate *WNT* signaling and variable RA receptor genotypes. HT29 and SW480 cells have mutations in *APC*, and HCT116 cells have mutations in beta-catenin (*CTNNB1*). HT29 cells have wild-type RA receptors, while HCT116 cells have mutations in *RARA*, and SW480 cells have mutations in *RARA* and *RXRG*.

The combinations of Sulindac and Piroxicam with 9-cis-RA, Liarozole, and Talarozole showed different CRC cell responses. HCT116 cells produced synergistic effects with 9-cis-RA + Sulindac and Piroxicam, Liarozole + Piroxicam, Talarozole + Sulindac, and Piroxicam, and showed additivity with Liarozole + Sulindac (Figure 1). In comparison, HT29 cells produced additive effects with 9-cis-RA + Sulindac, Liarozole + Sulindac, and Piroxicam (Figure S1). SW480 cells produced synergistic effects with Talarozole + Sulindac and additivity with 9-cis-RA + Sulindac and Liarozole + Sulindac, and Piroxicam (Figure S2).

**Figure 1.**
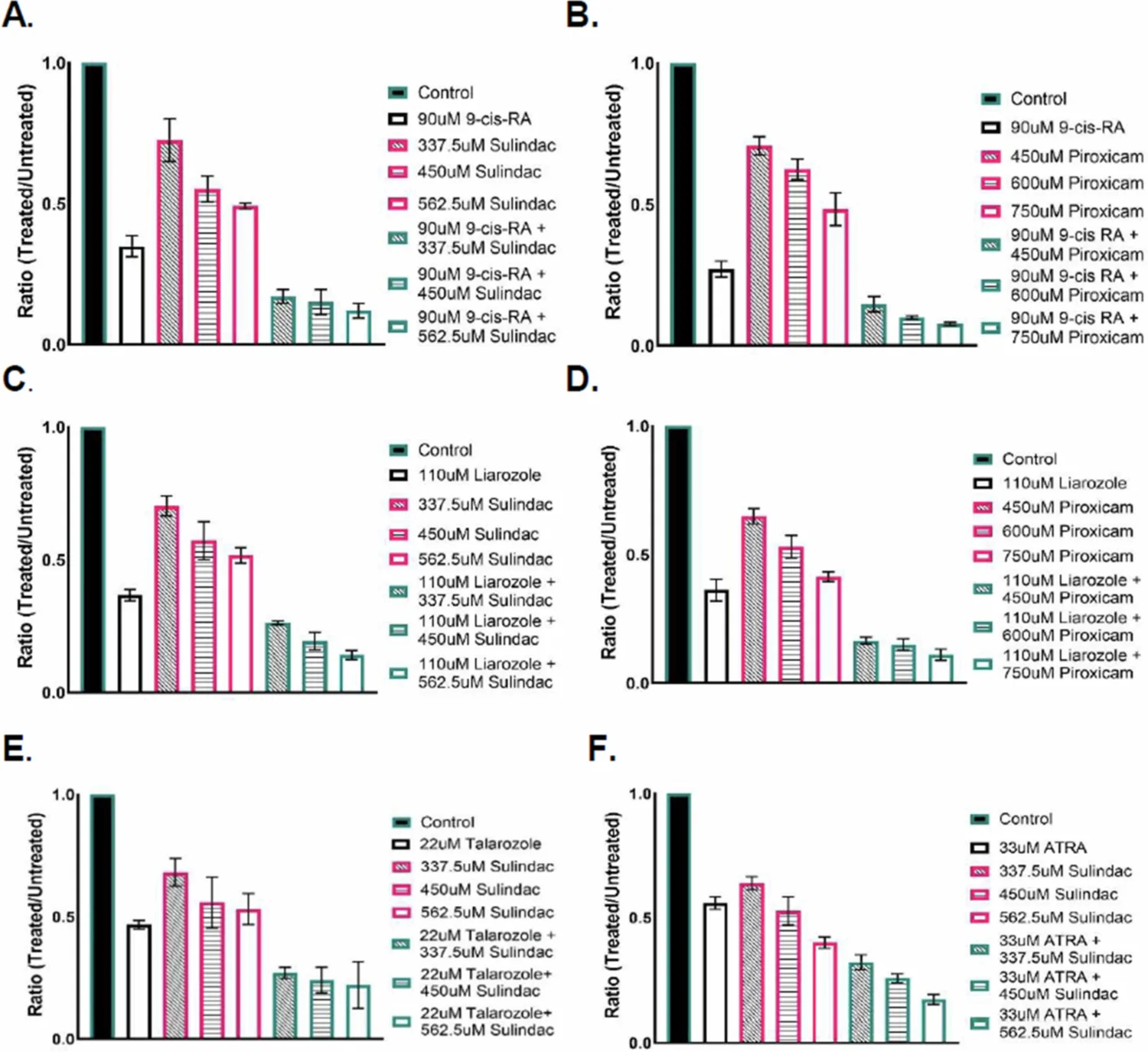
The combined effect of retinoid agents and anti-WNT agents on HCT116 cell proliferation. The specificity of drug response of the drugs administered as single agents or in combination was evaluated by cell counting using Trypan blue exclusion. The screening included combination experiments based on the IC50 dose at 48 h. As shown in (Panels A–F), there was a linear decrease in the response to increasing doses of the agents. This trend was observed across all the different drugs that were tested. This experiment was done in triplicate, and the error bars represent  ±  the SEM.

The response of drug combinations was further evaluated for antagonistic, additive, and synergistic responses on CRC cell proliferation (Figure 2). Figure 2A shows that 9-cis-RA + Sulindac and Piroxicam, Liarozole + Piroxicam, and ATRA + Sulindac have additive effects in HT29 cells. Figure 2B shows that 9-cis-RA + Sulindac and Piroxicam, Liarozole + Piroxicam, and ATRA + Sulindac have synergistic effects in HCT116 cells. Figure 2C shows that Talarozole + Sulindac have synergistic effects in SW480 cells. Table 1 summarizes the results on drug combinations that show additive and synergistic CRC cell responses.

**Figure 2.**
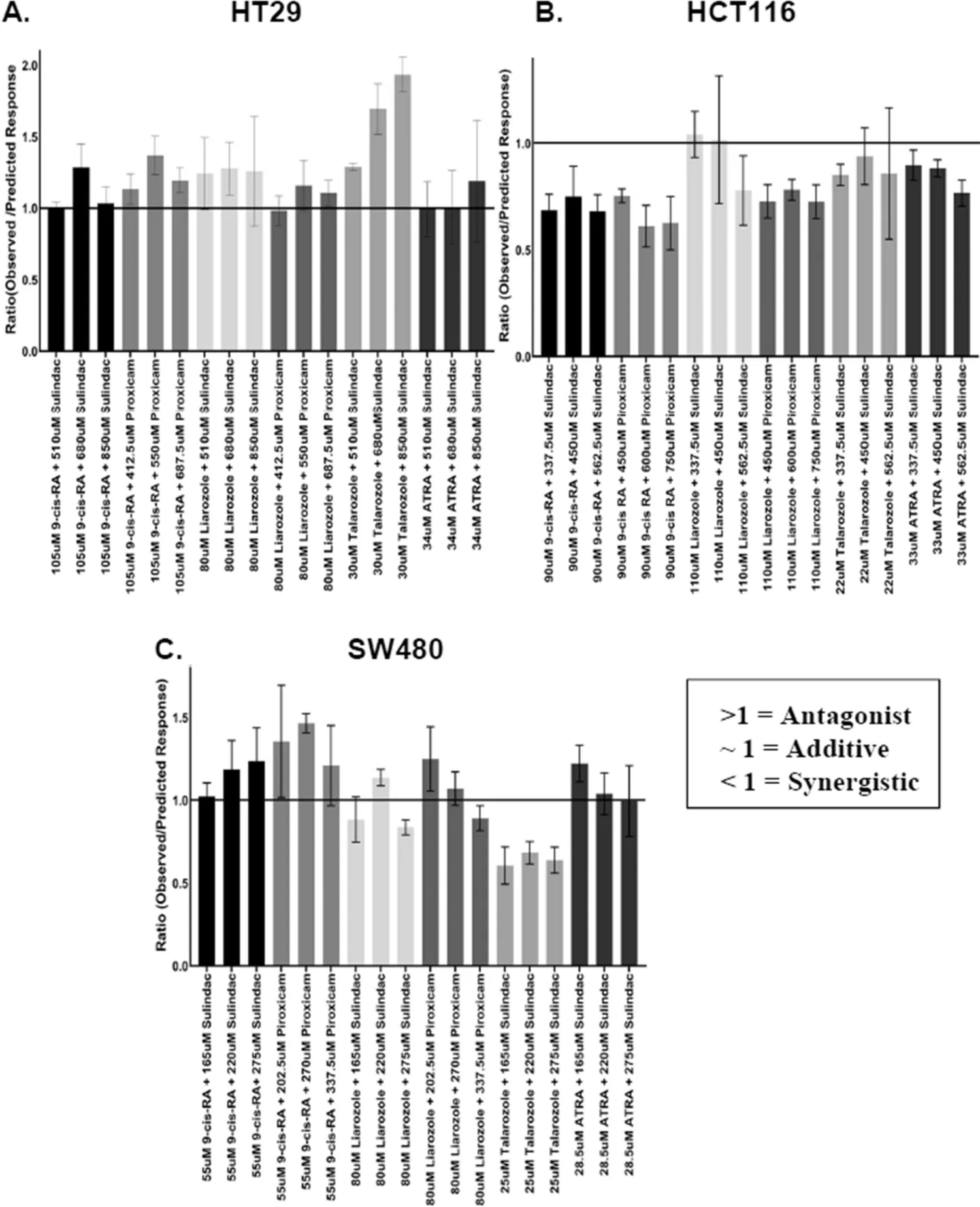
Evaluation of the drug combinations for anti-proliferative effects on different CRC cell lines. The response of drug combinations was further evaluated for antagonistic, additive, and synergistic responses on CRC cell proliferation. The combination effects were calculated as a ratio of the observed response divided by the predicted response based on the Bliss independence model.^52^ Panel A shows that 9-cis-RA + Sulindac and Piroxicam, Liarozole + Piroxicam, and ATRA + Sulindac have additive effects in HT29 cells. Panel B shows that 9-cis-RA + Sulindac and Piroxicam, Liarozole + Piroxicam, and ATRA + Sulindac have synergistic effects in HCT116 cells. Panel C shows that Talarozole + Sulindac have synergistic effects in SW480 cells. The synergistic response is < 1, additive ~1, and antagonistic > 1.

**Table 1.** Drug combinations showing additive and synergistic CRC cell responses.

CRC cell line	9cis-RA + Sulindac	9cis-RA + Piroxicam	Liarozole + Sulindac	Liarozole + Piroxicam	Talarozole + Sulindac
**HT29**	Additive	─	Additive	Additive	─
**SW480**	Additive	─	Additive/Synergistic	Additive	Synergistic
**HCT116**	Synergistic	Synergistic	Additive/Synergistic	Synergistic	Synergistic

### Drug combinations decrease ALDH + SCs and increase NEC differentiation in CRC cells

To evaluate the effects of treatments of retinoids and anti-WNT agents on the proportion of ALDH + SC populations in CRC cell lines, we utilized the ALDEFLUOR assay that marks ALDH + SCs (Figure 3A-C). We found that 9-cis-RA and Sulindac substantially decreased the population of ALDH + SCs in HCT116 cells but not in HT29 or SW480 cells. Specifically, this effect was seen in HCT116 cells treated with 9-cis-RA + Sulindac at time points of 24 and 48 h. However, an upward trend was observed in HT29 and SW480 cells treated with 9-cis-RA + Sulindac. Western blotting was also used to evaluate the expression of NEC differentiation markers in CRC cells. The results (Figure 3D–I) showed that drug combinations increased NEC differentiation in HCT116 CRC cells, but not in SW480 CRC cells.

**Figure 3.**
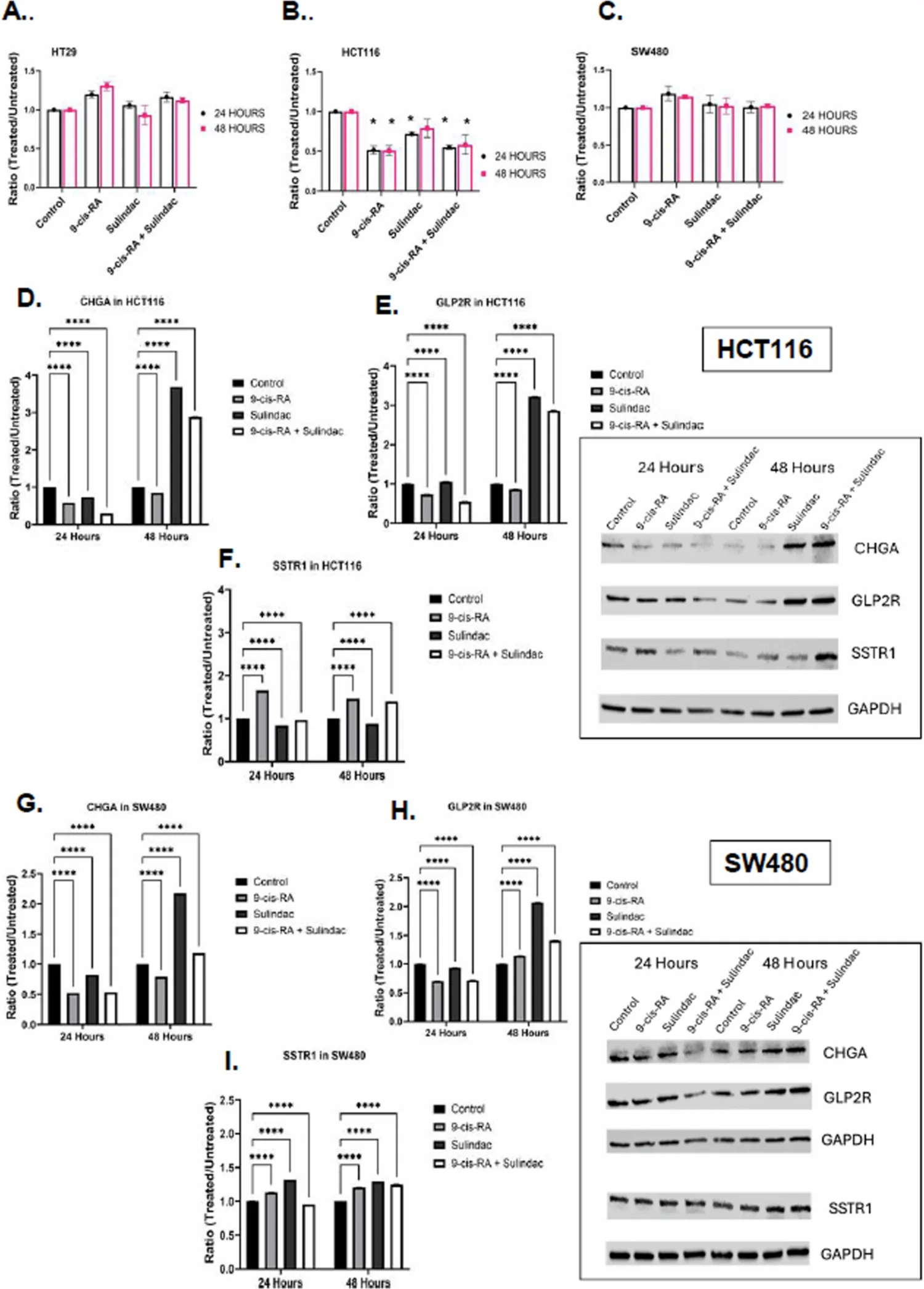
Drug combination effects on stem cells and neuroendocrine cells. The effects of retinoids and anti-WNT agents on ALDH + stem cells were evaluated based on the ALDEFLUOR assay. The results showed that treatment with the 9-cis RA + Sulindac drug combination substantially decreased the population of ALDH +  cells in HCT116 cells (Panel B), but not in HT29 (Panel A) or SW480 (Panel C) cells. Notably, this decrease was seen in HCT116 cells at both time points (24 and 48 h). *Neuroendocrine cells (NECs).* The effects of retinoids and anti-WNT agents on NEC differentiation were evaluated using western blot analysis for expression of differentiation markers in HCT116 CRC cells (Panels D–F) and SW480 cells (Panels G–I). The results show that drug combinations increase NEC differentiation in HCT116 and SW480 CRC cells. GLP2R = glucagon-like protein two receptors, SSTR1 = somatostatin receptor 1, CHGA = chromogranin A, NSE = neuron-specific enolase. The experiments were done in triplicate, and the error bars represent  ±  the SEM. * *p* < 0.05.

### NanoString profiling reveals that WNT inhibition alters RA-mediated gene expression

We conducted NanoString profiling of HCT116 and SW480 cells treated with the study agents to evaluate their effect on gene expression in WNT and RA signaling responses (Figure 4). The heatmap results (Figure 4A, B) show that the treatment of CRC cells with single agents and drug combinations leads to dramatic differences in gene expression between the different treatments. Further analyses showed that Sulindac treatment leads to changes in the expression of a substantial number of genes (Figure 4C, D). More genes were down-regulated than up-regulated (>2*X*-fold) by Sulindac in both HCT116 and SW480 cells. Changes in the expression of genes with WNT-regulatory elements in HCT116 (*n* = 23) and SW480 (*n* = 29) cells indicate that Sulindac modulates the expression of WNT-target genes (Figure 4E, F).

**Figure 4.**
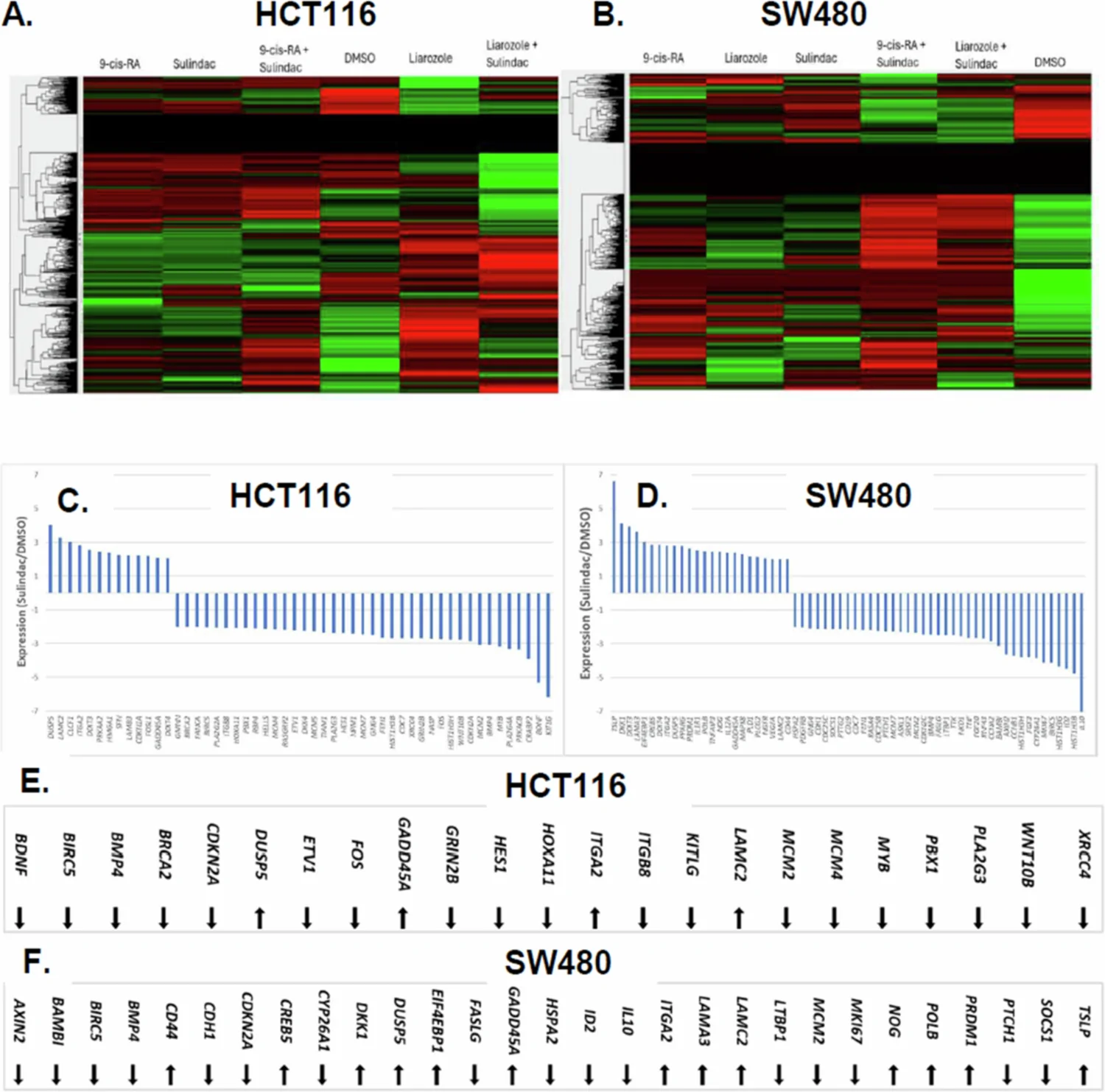
The effect of drug combinations on changes in gene expression in CRC cells. We conducted NanoString Profiling of HCT116 and SW480 cells treated with 9-cis-RA and Liarozole in combination with Sulindac to study the effects of drug combinations on gene expression. The heat maps (Panels A & B) show that drug combination treatments involving an RA agent (9-cis RA or Liarozole) in combination with an anti-WNT agent (Sulindac) lead to dramatic differences in gene expression compared to treatment with single agents. Sulindac treatment leads to the downregulation of a substantial number of genes in HCT116 cells (Panel C) and SW480 cells (Panel D). This down-regulation is characterized by changes in the expression of various *WNT* target genes. Changes in the expression of genes with WNT-regulatory elements (WREs) in HCT116 (Panels A and C) and SW480 (Panels B and D) cells indicate that Sulindac targets WNT signaling. Genes with WNT-regulatory elements show up- or down-regulation of expression (>2X-fold) in response to Sulindac treatment in HCT116 (Panel C) and SW480 (Panel D) CRC cells. In HCT116 cells, decreased expression of the notable WNT target genes *MET*, *MYC*, *AXIN2*, *BMP4*, *RNF43*, and *TCF3* are shown (Panel E). While in SW480 cells, the expression of the WNT target genes *AXIN2*, *BMP4*, *RNF43*, and *CCND1* had decreased (Panel F).

We also evaluated the effect of 9-*cis* retinoic acid and Liarozole on gene expression. NanoString mRNA profiling shows that similar genes are targeted by both of these retinoid agents in HCT116 and SW480 CRC cells (Venn diagrams, Figure 5A, B). Indeed, 19 of the same genes changed in expression (7 upregulated and 12 down-regulated) with both RA agent treatments (9-*cis* RA & Liarozole) and both CRC cell lines (Figure 5C).

**Figure 5.**
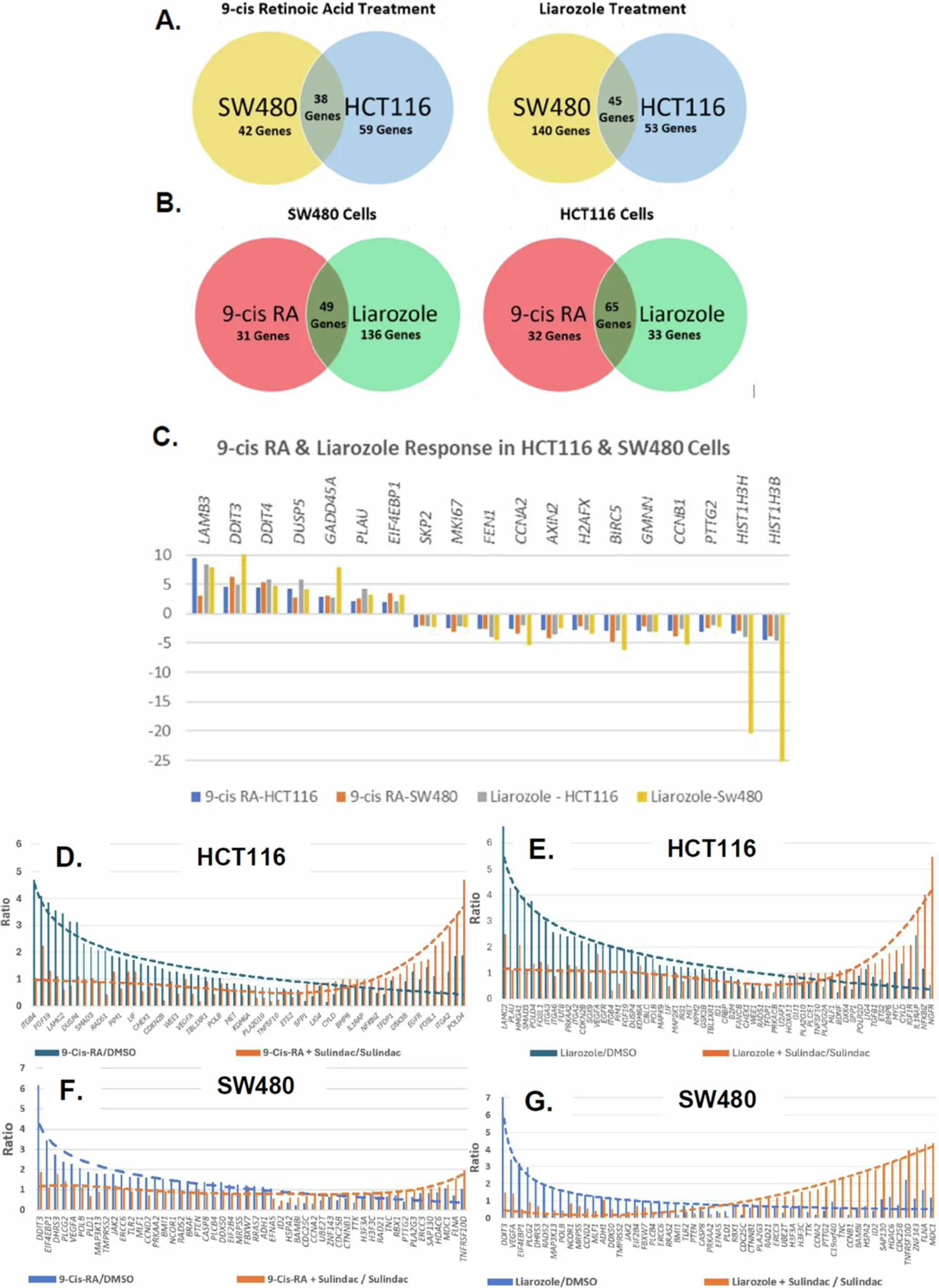
Analysis of Sulindac’s ability to sensitize CRC cells to the effect of retinoids based on gene expression. Further NanoString expression profiling was done on HCT116 and SW480 cells treated with 9-cis-RA or Liarozole in combination with Sulindac. *The effect of RA agents on CRC cells*. The Venn diagrams (Panel A) show that changes in the expression (*p* < 0.05) of the same genes in both cell lines (HCT116 and SW480) occurred with 9-cis-RA treatment (*n* = 38 genes) and Liarozole treatment (*n* = 45 genes). The Venn diagrams (Panel B) also show that changes in the expression (*p* < 0.05) of the same genes occur with both RA agents (9-cis-RA and Liarozole) in HCT116 (*n* = 49 genes) and SW480 cells (*n* = 65 genes). These results indicate that a common set of genes is targeted by both of the RA agents (9-cis-RA and Liarozole) in CRC cells. Indeed, 19 of the same genes changed in expression (7 upregulated and 12 down-regulated) with both RA agent treatments and in both CRC cell lines (Panel C). *The effect of Sulindac on responses to RA agents*. In both HCT116 (Panels D and E) and SW480 (Panels F and G) cells, one set of genes is down-regulated, and another set is up-regulated (*p* < 0.05) by retinoid agents after Sulindac treatment. Additional analyses to illustrate the change in the expression of the specific genes are presented in waterfall plots (Figure S3) and in lists of the top 10 genes upregulated (Table S3) and downregulated (Table S4) by RA agents after Sulindac treatment. Interestingly, many of the same genes are upregulated or downregulated by Liarozole and 9-cis RA in both cell lines. Overall, these results indicate that Sulindac sensitizes HCT116 and SW480 CRC cells to the effect of retinoids on gene expression.

We then compared the effect of retinoids versus Liarozole in combination with Sulindac on gene expression (Figure 5D-G). We found that two sets of genes are differentially modulated by retinoid agents after CRC cells are treated with Sulindac. In both HCT116 (Figure 5D, E) and SW480 (Figure 5F, G) cells, one set of genes is down-regulated, and another set is up-regulated by retinoid agents after Sulindac treatment. These results indicate that decreasing WNT signaling by Sulindac sensitizes CRC cells to the effect of retinoids on gene expression.

Further study using David Annotation Analysis shows that the two gene sets that are up-regulated versus down-regulated have different functional classifications (Table 2). Also, bioinformatics analyses based on KEGG pathway enrichment (Figure S4) reveal that the majority of these genes are involved in pathways that regulate cell proliferation as well as growth suppression and differentiation. Since Sulindac modulates the expression of genes with WREs, and 9-cis RA & Liarozole both target a similar set of genes, this indicates that anti-WNT agents and RA-agents function through different mechanisms. Thus, the difference in gene response between anti-WNT and RA agents begins to explain how combinations of these drugs can produce synergistic effects and how Sulindac can sensitize CRC cells to the growth-inhibitory effects of retinoids.

**Table 2.** The two sets of retinoid-response genes (down-regulated *vs.* up-regulated) have different functional classifications.*

Cell line	Treatment	Down-regulated gene set	Up-regulated gene set
SW480	9-cis RA ± Sulindac	Signal transduction	Cell cycle
SW480	Liarozole ± Sulindac	Signal transduction	Cell cycle
HCT116	9-cis RA ± Sulindac	Tissue development	Cell differentiation
HCT116	Liarozole ± Sulindac	Cell adhesion	Cell differentiation

^*^ Based on DAVID functional classification of genes that become responsive to retinoid agents after sulindac treatment.

*TOPflash reporter and western blots show Sulindac and Piroxicam decrease WNT signaling.* To further quantify WNT activity in HT29, HCT116, and SW480 cells, we used the TOPflash Reporter Assay, which measures luciferase activity to indicate the canonical Wnt/β-catenin signaling pathway activity in cells. We explored the ability of Sulindac and Piroxicam to downregulate WNT signaling. The TOP-flash analysis (Figure 6A–C) showed that Sulindac and Piroxicam down-regulate WNT signaling in all three CRC cell lines. Furthermore, western blot analysis (Figure 6D–K) of the effect of Sulindac on TCF target gene expression also shows the down-regulation of WNT signaling. Thus, treatment of CRC cells with Sulindac and Piroxicam significantly decreases WNT signaling activity, further demonstrating the efficacy of these agents in targeting WNT signaling.

**Figure 6.**
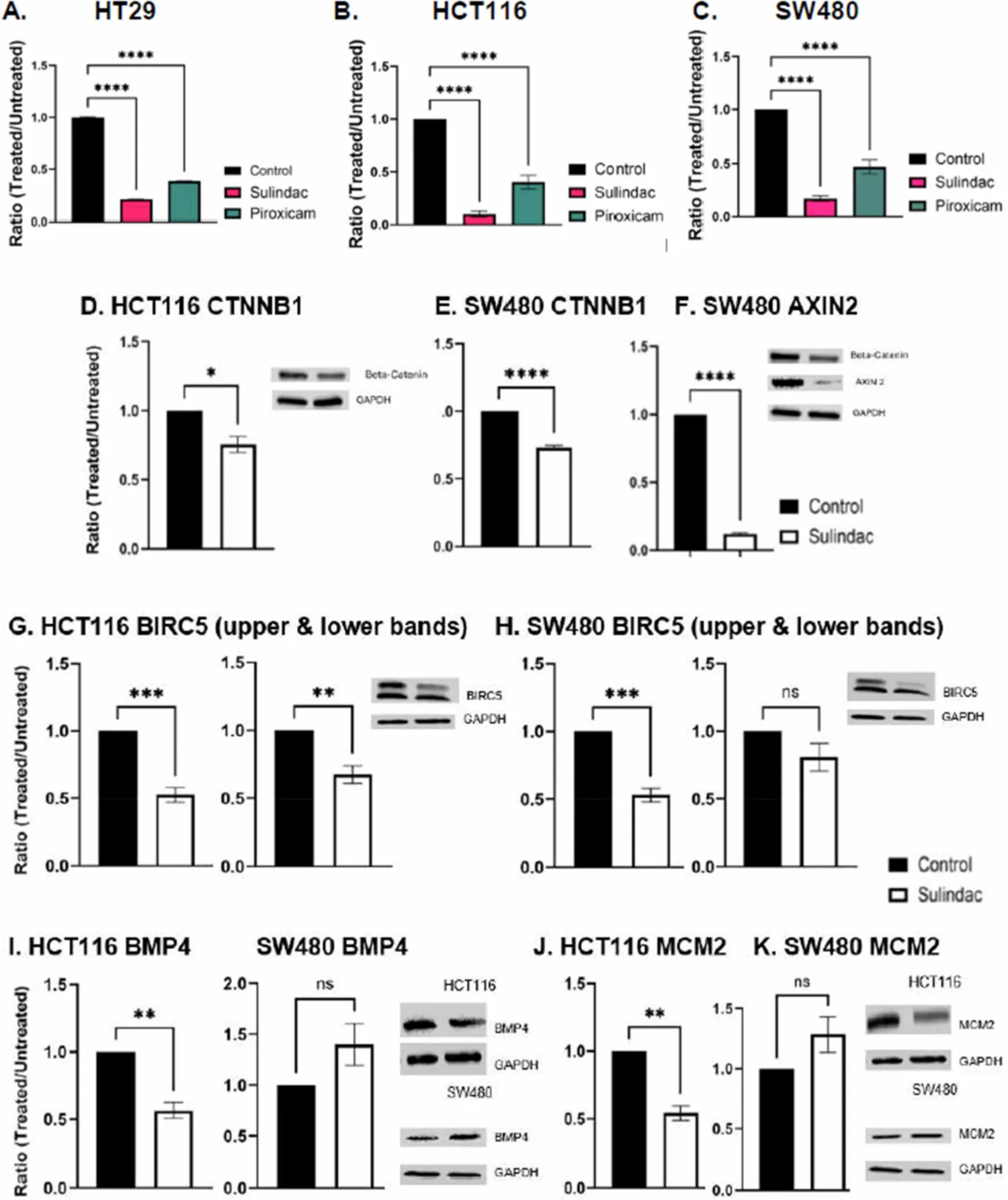
Sulindac modulates WNT signaling. *WNT signaling* . Sulindac and Piroxicam both reduced WNT/β-catenin activity in HT29, HCT116, and SW480 cells (panels A–C). WNT/β-catenin activity was measured using the TOPflash-based TCF/LEF reporter luciferase assay. Luciferase activity was measured, and the results were normalized to those of the untreated samples. This experiment was done in triplicate, and the error bars represent  ±  the SEM. **** *p* < 0.0001. *WNT target gene expression* *in HCT116 and SW480 cells*. Western blots and densitometry were used to evaluate the protein expression of WNT target genes. The results show that Sulindac treatment decreases β -catenin expression in HCT116 (Panel D) and SW480 cells (Panel E). A decrease in AXIN2 protein expression was observed in SW480 cells (Panel F). For BIRC5 (Panels G and H), a decrease in protein expression was observed in HCT116 cells (both upper & lower bands) and in SW480 cells (upper band). A decrease in BMP and MCM2 expression occurred with Sulindac treatment in HCT116 cells, but not in SW480 cells (Panels I–K). This process was done in triplicate, and the error bars represent  ±  the SEM. *p < 0.05, ** *p* < 0.01, *** *p* < 0.001, **** *p* < 0.000, ns – not significant.

### Treating CRC organoid cultures with drug combinations leads to greater anti-tumor activity as compared to individual agents

The effect of these drug treatments was further evaluated using a human CRC organoid model. Figure 7A and B show representative microscopic staining images based on cell viability analysis using the CYTO 3D Live/Dead Assay. This assay qualitatively shows that treating CRC organoid culture types with both drug combinations leads to greater anti-tumor activity as compared to individual agents. Quantitative analysis based on the PI (dead cells)/AO (live cells) ratio reveals that an increased proportion of dead cells to live cells occurs with the combination treatments as compared to individual treatments alone (Figure 7C, D).

**Figure 7.**
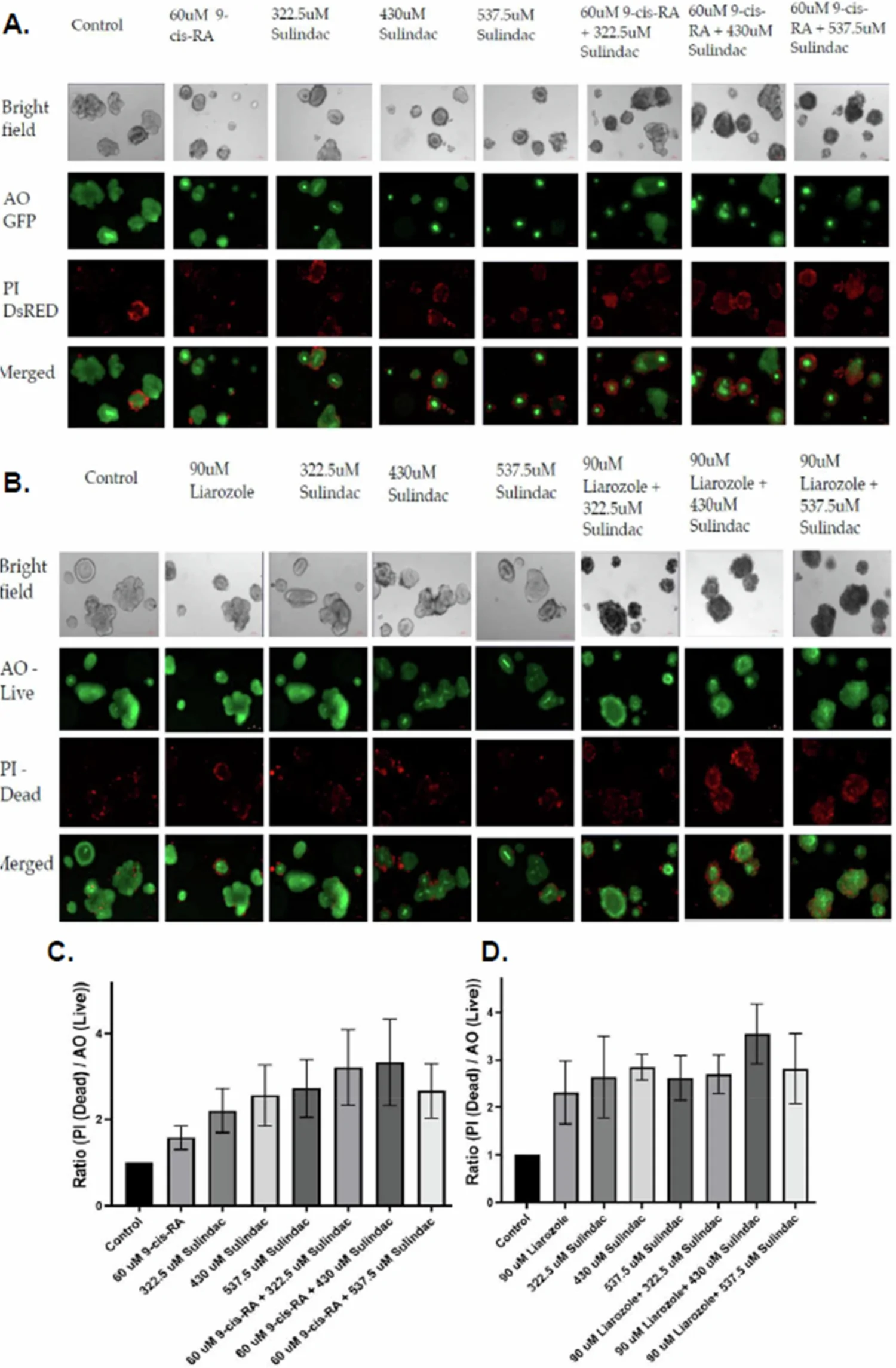
Measuring drug responses using organoid cultures of human CRC cells. The effect of therapeutic manipulation of WNT and RA signaling was evaluated in human patient-derived colon cancer organoids. Drug-response experiments were conducted using a CRC organoid model from the ATCC (HCM-CSHL-0257-C18). Treatment concentrations were chosen based on the effective IC50 doses as indicated: 9-cis-RA (90 µM), Sulindac (322.5, 430, 537.5 µM), and Liarozole (90 µM). Panels **A & B** show representative microscopy staining from cell viability analysis using CYTO 3D Live/Dead Assay Kit. Scale bars equal 50 µM. Each panel contains four rows of images. Brightfield shows the overall cell morphology under normal light. AO (acridine orange): green fluorescence indicating live cells. PI (propidium iodide): red fluorescence marks dead cells. Merged: A combination of AO and PI channels showing both live and dead cells simultaneously.Panels C and D give the quantification for cell death based on the PI (dead cells)/AO (live cells) ratio. The cells were treated with various concentrations of 9-cis-RA, Liarozole, Sulindac alone, or combinations of both compounds. The control represents untreated cells. Increasing ratios indicate higher proportions of dead cells than live cells, with combination treatments showing the most pronounced effects. This experiment was done in triplicate, and the error bars represent ± the SEM. Overall, treating CRC organoid cultures with anti-WNT & RA drug combinations causes a substantial increase in cell death compared to individual agents alone.

### Anti-tumor activity of drug combinations in a human CRC PDX mouse model

The drug effectiveness using a *CTNNB1*-mutant human CRC xenograft line grown in immunodeficient (*nu/nu* Nude) mice was also examined. Drug toxicity was initially evaluated for tolerability (Table S5) based on body weight measurements. Body weights remained mostly stable with no weight loss greater than 10% (Figure S5). The triple combination treatment group maintained slightly lower but stable body weights, supporting an acceptable treatment regimen.

The efficacy study evaluated treatment outcomes by monitoring the mice's body weight (Table S6, Figure 8A) and measuring the tumor volume (Figure 8B, Table 3). Regarding tolerability, absolute body weight measurements remained stable (22–28 g range), indicating general treatment tolerability, and percent change analyses showed positive weight gain (3%–13%) across most groups, implying minimal systemic toxicity. The triple combination treatment group maintained slightly lower, but stable body weights, supporting an acceptable treatment regimen.

**Figure 8.**
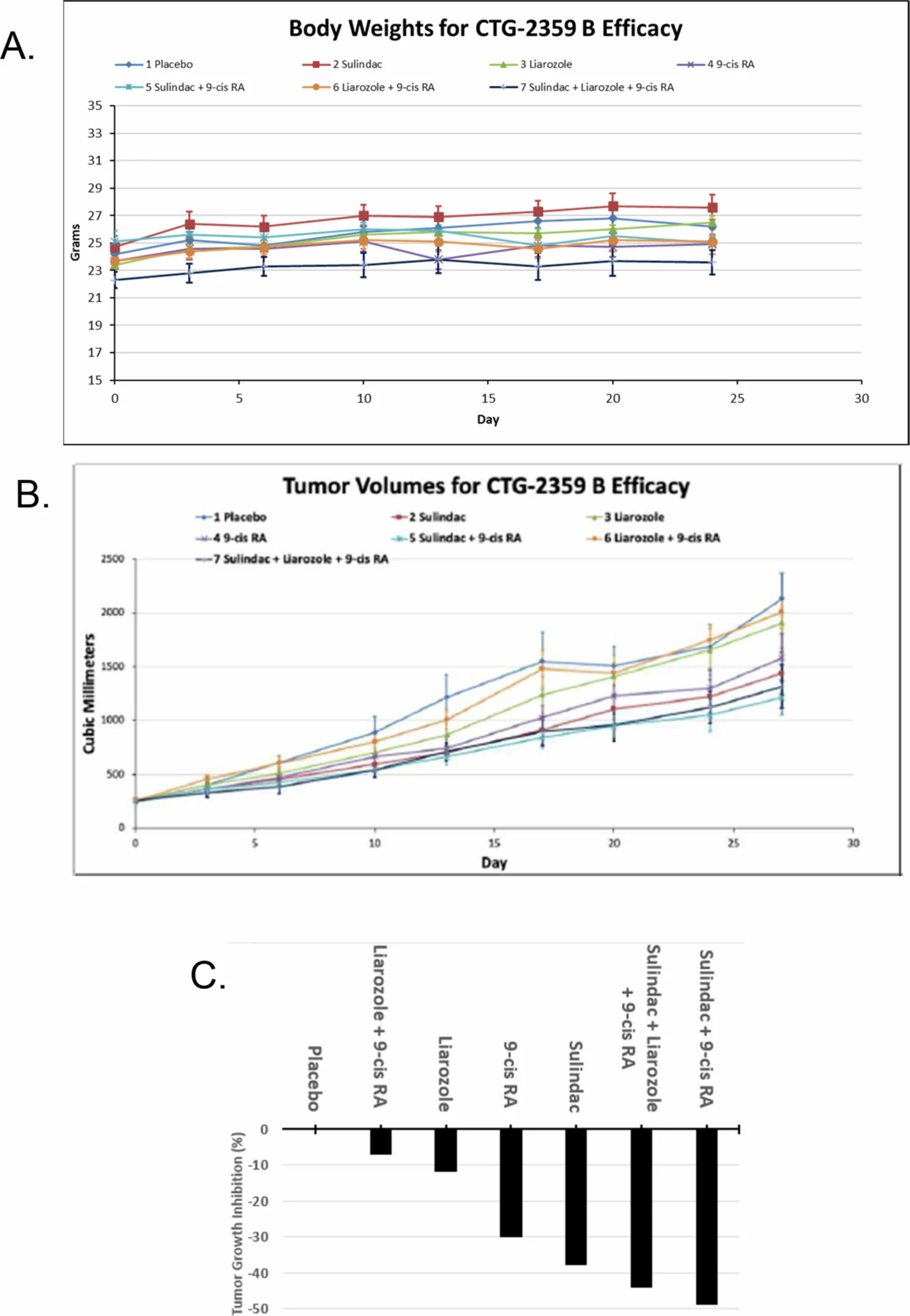
Drug combination responses in a human CRC PDX (CTG-2359) mouse model. *Drug safety*. Drug toxicity was initially evaluated for tolerability (Panel A), and during drug treatments for efficacy. Different colored lines indicate various treatment conditions, including control and combination treatments. Body weights remained mostly stable with no weight loss greater than 10%. This reveals that the drug treatments were reasonably well tolerated.*Efficacy analysis*. Panel B shows the time course results on tumor volume changes in the efficacy study over 28 d. The treatment groups included a placebo control and various Sulindac, Liarozole, and 9-cis RA combinations. The tumor volumes were measured in cubic millimeters and plotted against time (days). The error bars represent ± the SEM. All the treatment groups showed progressive tumor growth, with the combinations of Sulindac + Liarozole + 9-cis RA demonstrating the lowest tumor volume trajectory. The placebo group exhibited the highest tumor growth rate, reaching approximately 2000 mm^3^ by day 25. Measurements were taken at 5-day intervals after treatment initiation at day 0. Panel C. The waterfall plot shows the percent decreased in tumor growth for all treatment groups. The corresponding values for anti-tumor activity are provided in Table 3 below.

**Table 3.** Anti-tumor activity for human CRC PDX (CTG-2359) mouse model.

Treatment	Mean ± SEM (mm^3^)	*p*-value *vs*. control	%TGI
Placebo	2135 ± 237	–	–
Sulindac	1438 ± 195	0.0969	38
Liarozole	1910 ± 233	0.9249	12
9-cis RA	1584 ± 220	0.2785	30
Sulindac + 9-cis RA	1214 ± 164	0.0159	49
Liarozole + 9-cis RA	2009 ± 146	0.9959	7
Sulindac + Liarozole + 9-cis RA	1318 ± 201	0.0384	44

The raw tumor volume measurements indicated continuous growth across all treatment groups, with final volumes ranging from approximately 1000–2000 mm^3^ by day 25 (Table 3). The Log10-transformed data revealed a linear growth pattern of the placebo control (Figure S6), showing that tumor growth followed exponential kinetics. The triple combination treatment (Sulindac + Liarozole + 9-cis RA) demonstrated the lowest absolute tumor volumes throughout the entire study period. Single-agent treatments (Sulindac, Liarozole, 9-cis RA) resulted in higher tumor volumes, while demonstrating good tolerability with positive weight gain trends. Overall, the correlation between treatment efficacy and tolerability showed that the triple combination treatment provided the best response (Figure 8A-C), and it also maintained an acceptable therapeutic window. In conclusion, the study indicates that the triple combination treatment therapy is the most promising treatment option, balancing efficacy and tolerability, with all treatments being generally well-tolerated based on stable or increasing body weights.

## Discussion

The goal of our study was to sensitize human colorectal cancer (CRC) to the differentiation-inducing effects of retinoic acid (RA) agents by suppressing WNT signaling. The main finding was that inhibiting WNT signaling effectively sensitizes CRC cells to the anti-tumor effects of retinoids. Specifically, treating human CRC cell lines, organoid cultures, and a PDX mouse model with drug combinations leads to greater anti-tumor activity as compared to individual agents. To investigate the underlying mechanisms, we used several independent experimental approaches to determine how the inhibition of WNT signaling sensitizes CRC cells to the anti-tumor effects of retinoids. Our findings from NanoString profiling, western blot analysis for cell differentiation, and quantification of SC populations further support our hypothesis that therapeutically inhibiting WNT signaling enhances the ability of retinoid agents to induce growth suppression and differentiation. The following points bear further discussion.

1) *How does the administration of anti-WNT agents sensitize CRC cells to the anti-proliferative and anti-tumor effects of RA agents?*

Showing that anti-WNT agents Sulindac and Piroxicam sensitize CRC cells to the anti-proliferative and anti-tumor effects of RA agents raises the question: What is the underlying mechanism? The answer relates to the fact that we found that WNT and RA signaling are linked in CRC^23^ and that constitutively activated WNT signaling drives the growth of most (~90%) CRCs. When WNT signaling is constitutively activated by *APC* or *CTNNB1* mutation, it leads to incomplete differentiation during CRC development. Since RA signaling plays a crucial role in inducing cellular differentiation, the activated WNT signaling leads to an inability of RA to induce cellular differentiation. Thus, suppressing WNT signaling with anti-WNT agents such as Sulindac and Piroxicam allows the ability of RA agents to induce cellular differentiation. Indeed, we showed that Sulindac and Piroxicam decrease WNT signaling using: 1) TOPflash WNT reporter assay and 2) expression of WNT target genes (Figure 3). Moreover, our results (Figures 1 and 2) show that a decrease in WNT signaling sensitizes CRC cells to the activation of RA signaling in response to RA agents or by the inhibition of RA metabolism.

2) *Why do drug combinations decrease the proportion of ALDH + SCs and increase NEC differentiation in HCT116 cells, but not SW480 CRC cells?*

In our evaluation of the effects of treatments of retinoids and anti-WNT agents on the proportion of ALDH + SC populations in CRC cell lines, we observed that 9-cis-RA and Sulindac substantially decrease the population of ALDH +  cells in HCT116 cells but not in HT29 and SW480 cells (Figure 4). The mechanism may be due to the difference in the activation of WNT signaling in these cell lines. In HCT116 cells, WNT signaling is activated by *CTNNB1* mutation. In HT29 and SW480 cells, WNT signaling is activated by *APC* mutation. Since we and others have shown that Sulindac downregulates WNT signaling by inducing the degradation of beta-catenin in CRC cells,^35‐46^ it indicates that Sulindac may have a greater effect on HCT116 cells with *CTNNB1* mutation than on HT29 and SW480 cells with *APC* mutation. Moreover, Piroxicam, which is another WNT inhibitor,^47‐51^ had a superior effect on HCT116 cells than HT29 and SW480 cells (Figure 2, Table 1).

Nonetheless, we tested to see if Sulindac affects WNT signaling in SW480 cells with mutant *APC* by analyzing its ability to decrease the expression of WNT target genes. Indeed, we found, using mRNA NanoString profiling and western blot analysis, that Sulindac administration decreases the expression of WNT target genes in SW480 cells. We also analyzed whether Sulindac is able to sensitize SW480 cells to the differentiation-inducing effects of RA signaling. Previously, we showed that ALDH and other RA pathway components are selectively expressed in ALDH + colonic SCs, which indicates that RA signaling mainly occurs in ALDH + SCs.^24^ We also found that ATRA-treated CRC cells have reduced proliferation and increased SC differentiation along the NEC lineage.^23^ ^,^ ^25^ ^,^ ^53^ ^,^ ^55^ Thus, we surmised that if Sulindac was able to decrease WNT signaling in SW480 cells, it should sensitize them to RA-induced differentiation along the NEC lineage. However, unlike in HCT116 cells, we found that Sulindac, when combined with 9-cis RA, did not increase NEC differentiation.

This finding might suggest that Sulindac is more effective against *CTNNB1* mutant tumors and less effective against *APC* mutant tumors. Nonetheless, we did find that drug combinations involving 9cis-RA + Sulindac, Liarozole + Sulindac, Liarozole + Piroxicam, and Talarozole + Sulindac produced additive or synergistic effects on SW480 and HT29 cell proliferation (Table 1). Moreover, Sulindac is clinically effective in preventing and shrinking adenomas in the treatment of FAP patients who carry germline *APC* mutations.^42‐46^ To sort out this apparent discrepancy between the drug sensitivity of *CTNNB1* and *APC* mutant tumors, we performed NanoString mRNA profiling to assess the effects of drug combinations on gene expression in HCT116 cells versus SW480 CRC cells.

3) *How does gene expression change in response to RA treatment when CRC cells are treated with anti-WNT agents?*

We performed NanoString mRNA profiling to assess the effects of RA and WNT agents on gene expression when they are administered as single agents or in combination. When Sulindac or Piroxicam were delivered as single agents, they significantly decreased the expression of a number of genes, and in both cases, many of the genes were WNT-target genes. We then evaluated which genes were significantly changed in expression in both experiments involving 9-cis RA + Sulindac versus Liarozole + Sulindac and then found that many of these genes were the same genes between these two sets. Interestingly, we found that the change in direction of gene expression (up or down) was the same for all the genes in this set of common genes. We then analyzed expression patterns in the individual experiments (9-cis RA + Sulindac versus Liarozole + Sulindac) to find differences in how HCT116 cells respond differently to drug combinations compared to SW480 cells. We did this by comparing the effect of single agent 9-*cis* RA versus 9-*cis* RA + Sulindac, as well as the single agent Liarozole versus Liarozole + Sulindac, to determine how the Sulindac affects the response to RA agents. We found that when 9-cis RA and Liarozole are administered as single agents, a specific expression pattern of upregulated and downregulated genes occurs, but when Sulindac is administered, the effect of 9-cis RA and Liarozole change by reversing this pattern of gene expression. This trend in the suppression of the effects was similar in both cases when Sulindac was combined with 9-cis RA or Liarozole. However, the specific sets of genes that were undergoing these changes were different between the two experiments involving HCT116 cells versus SW480 cells. Thus, these differences in gene expression in response to anti-WNT agents administered in combination with RA agents may explain the differences between HCT116 cells and SW480 cells’ responses involving SCs and the differentiation described in Section 2 above.

4) *Do the results provide adequate evidence for anti-tumor efficacy of the drug combination*?

Our study provides three independent assays for the efficacy of drug combinations that target WNT and RA signaling. For HT29, HCT116, and SW480 cells, all three cell lines showed that the treatment, both as a single agent and in combinations, decreased proliferation in a dose- and time-dependent manner. However, only HCT116 cells showed that the majority of drug combinations were synergistic. Since HCT116 cells have a *CTNNB1* mutation and HT29 and SW480 cells have *APC* mutations that lead to activated WNT signaling, this finding indicates that Sulindac is more effective against CRCs with a *CTNNB1* mutation than those that have *APC* mutations. This concurs with reports that we and others have shown that Sulindac downregulates WNT signaling by inducing the degradation of beta-catenin in CRC cells.^35‐39^ Further testing for efficacy involving treatments with 9-*cis*-RA, Liarozole, and Sulindac led to increased cell death rates in organoid cultures and was effective by decreasing tumor size in PDX mice. Overall, this study's findings support the hypothesis that therapeutically inhibiting WNT signaling enhances the ability of retinoid agents to induce growth suppression and differentiation of CRC cells.

## Conclusion

Understanding how to target the imbalance between WNT and RA signaling in CRC offers great promise for developing novel therapeutic strategies that restore normal tissue homeostasis. Our study explored combination therapies that target both WNT and RA signaling, which embraces the idea of RA-based differentiation therapies for the treatment of CRC and other cancers.^2‐8^ Our approach differs from conventional systemic cytotoxic and immunologic types of cancer therapies by understanding the mechanisms that normally control cell numbers in colonic epithelium. In the normal colon, this mechanism is based on a precisely-controlled balance between cell proliferation and differentiation.^56^ ^,^ ^57^ Maintaining this balance relies on two main pathways, WNT signaling and retinoic acid signaling.^23^ ^,^ ^58^ Since *APC* and *CTNNB1* are driver mutations that constitutively activate WNT signaling, which inhibits retinoic acid signaling, we developed drug combinations to reverse the effects of WNT activation and to sensitize CRCs to the anti-tumor effects of retinoids. Indeed, our combination of anti-WNT agents and retinoid drugs effectively restored the ability of retinoids to induce growth suppression and differentiation of CRC tissues. Our findings also hold promise for future studies in precision oncology because many new anti-WNT agents are now emerging in clinical trials or are under investigation.^33^ These findings provide a basis for therapeutically reducing the number of cell divisions that tumor cells can undergo by inducing their differentiation. This mechanistic-based treatment approach provides an innovative way to restore tissue homeostasis and control tumor growth rather than just eliminating cancer cells.

## Supplementary Material

Supplemental Material Supplemtary_Materials_vRevised_final.docx

Supplemental Material Arrive_10_2_parts.pdf

## Data Availability

The data underlying this article will be shared on reasonable request to the corresponding author.
