# Supplementary material for: Inhibition of WNT signaling sensitizes CRC cells to anti-tumor effects of retinoids: basis for new drug combinations in oncology
Source: Cancer Biol Ther. 2026 Oct 8;27(1):2732738. doi: 10.1080/15384047.2026.2732738 (PMC13652067; doi:10.1080/15384047.2026.2732738)
Supplement: Supplementary file 2 [file KCBT_A_2732738_SM1233.docx]

**Supplementary Materials**

**A**	**B**

**C**	**D**

**E**	**F**

**Figure S1:** **The combined effect of retinoid agents and anti-WNT agents on HT29 cell proliferation.** The specificity of drug response of the drugs administered as single agents or in combination was evaluated by cell counting using Trypan blue exclusion. The screening included combination experiments based on the IC50 dose at 48 hrs. As shown in (Panels **A-F**), there was a linear decrease in the response to increasing doses of the agents and combination agents. This trend was observed across all the different drugs that were tested. This experiment was done in triplicate, and error bars represent ± the SEM.

A.	B.

C.	D.

E.	F.

**Figure S2: The combined effect of retinoid agents and anti-WNT agents on SW480 cell proliferation.** The specificity of drug response of the drugs administered as single agents or in combination was evaluated by cell counting using Trypan blue exclusion. The screening included combination experiments based on the IC50 dose at 48 hrs. As shown in (Panels **A-F**), there was a decrease in the response to combination agents. This experiment was done in triplicate, and error bars represent ± the SEM.

**Figure S3: Waterfall plots of the response of CRC cells to administration of retinoid agents after Sulindac treatment.**

**Panel A. Top enriched KEGG pathways for genes downregulated by RA agents (Liarozole & 9-cis RA) after Sulindac treatment of HCT116 cells.**

**Panel B. Top enriched KEGG pathways for genes upregulated by RA agents (Liarozole & 9-cis RA) after Sulindac treatment of HCT116 cells.**

**Panel C. Top enriched KEGG pathways for genes downregulated by RA agents (Liarozole & 9-cis RA) after Sulindac treatment of SW480 cells.**

**Panel D. Top enriched KEGG pathways for genes upregulated by RA agents (Liarozole & 9-cis RA) after Sulindac treatment of SW480 cells.**

**Figure S4. Top enriched KEGG pathway analysis.** Panel A. Enriched pathways for genes downregulated by RA agents (Liarozole & 9-cis RA) after Sulindac treatment of HCT116 cells. Panel B. Enriched pathways for genes upregulated by RA agents (Liarozole & 9-cis RA) after Sulindac treatment of HCT116 cells. Panel C. Enriched pathways for genes downregulated by RA agents (Liarozole & 9-cis RA) after Sulindac treatment of SW480 cells. Panel D. Enriched pathways for genes upregulated by RA agents (Liarozole & 9-cis RA) after Sulindac treatment of SW480 cells.

**Table 1. Molecular mechanisms for the anti-neoplastic effects of Sulindac and Piroxicam that are independent of COX2.**

**Sulindac:**

- Downregulates nuclear beta-catenin localization and beta-catenin/TCF-target gene transcription [40, 48, 61-67]
- Diminishes the expression of the protein Bcl-XL and the Bcl-XL antagonist of Bax to induce activation of caspase cascade, which stimulates the apoptosis process [68].
- Attenuates expression of the antiapoptotic protein survivin in CRC cells [36].
- Induces caspase- and proteasome-dependent degradation of beta-catenin protein in human CRC cells [37].
- Down-regulates Akt phosphorylation and inhibiting MMP-2 production [69, 70].
- Binds to the DVL-PDZ domain and directly inhibiting DVL’s interaction with FZDs [71].
- Inhibits of phosphodiesterase 5 (PDE5) to induce apoptosis [63, 72].
- Inhibits cyclic guanosine 31,51-monophosphate phosphodiesterase (cGMP PDE), increasing cGMP and activated cGMP-dependent protein kinase (PKG), which decreases transcriptional suppression of beta-catenin and inhibition of the WNT/ beta-catenin signaling [73].
- Decreases expression of the Bcl2 antiapoptotic protein in adenomas of Min mice [38].
- Exerts a dominant-negative effect upon intestinal cell migration [39].
- Suppresses NF‑κB activation, reducing the expression of pro‑survival and pro‑inflammatory genes [43].
- Decreases the activities of phosphatidylinositol-specific phospholipase C and levels of prostaglandin E2 [47].

**Piroxicam:**

- Suppresses WNT beta-catenin signaling [49-52].
- Inhibits plasma membrane fluidity [49].
- Increases calpain-9 and PPARγ expression [49].
- Reverses DNA hypomethylation in tumors [66].

| **Table S2:** List of primary and secondary antibodies. | | | |
| --- | --- | --- | --- |
| **Antibody** | Titer | Catalog # | Company |
| CHGA | 1:1000 | RQ5334 | NSJ BIOREAGENTS |
| GLP2R | 1:1000 | PA5-104301 | Invitrogen |
| SSTR1 | 1:1000 | bs-1137R | Bioss ANTIBODIES |
| GAPDH | 1:1000 | ab181602 | Abcam |
| CTNNB1 | 1:1000 | 8480 | Cell Signal Technologies |
| AXIN2 | 1:1000 | MA5-15015 | Invitrogen |
| BIRC5 | 1:1000 | NB500-201SS | Novus Biologicals |
| BMP4 | 1:1000 | MA5-15572 | Invitrogen |
| MCM2 | 1:1000 | 12079S | Cell Signaling |
| **Secondary antibody** | | | |
| m-IgGkBP-HRP (anti-mouse) | 1:1000 | sc-516102 | Santa Cruz Technologies |
| mouse anti-rabbit IgG-HRP | 1:1000 | Sc-2357 | Santa Cruz Technologies |

| **Table S3. Top ten genes upregulated by RA agents after Sulindac treatment** | | | |
| --- | --- | --- | --- |
| **HCT116 -Liarozole** | **HCT116 – 9-cis RA** | **SW480 - Liarozole** | **SW480 – 9-cis RA** |
| *NGFR* | *BDNF* | *CCNB1* | *CCNB1* |
| *BDNF* | *NGFR* | *CCNA2* | *BAMBI* |
| *NFKBIZ* | *SPP1* | *ID2* | *TTK* |
| *SPP1* | *POLD4* | *CDC25B* | *CCNA2* |
| *BMP6* | *DKK4* | *TTK* | *ID2* |
| *IGF1R* | *ITGA2* | *CDC25C* | *PTTG2* |
| *PRKACB* | *PRKACB* | *PTTG2* | *FLNA* |
| *DKK4* | *CYLD* | *UBE2T* | *CDC25C* |
| *PLA2G2A* | *NFKBIZ* | *HSPA2* | *TNFRSF10D* |
| *ETS2* | *FOSL1* | *BAMBI* | *CDC25B* |

| **Table S4. Top ten genes downregulated by RA agents after Sulindac treatment** | | | |
| --- | --- | --- | --- |
| **HCT116 -Liarozole** | **HCT116 – 9-cis RA** | **SW480 - Liarozole** | **SW480 – 9-cis RA** |
| *ITGB4* | *CDKN2B* | *DDIT3* | *DDIT3* |
| *DUSP4* | *MAPK9* | *TMPRSS2* | *EIF4EBP1* |
| *PLAU* | *LAMC2* | *RAD52* | *PLD1* |
| *SMAD3* | *RAD51* | *EIF4EBP1* | *CCND2* |
| *ID1* | *ITGB4* | *BMI1* | *PLCB4* |
| *PIM1* | *ITGA3* | *POLB* | *MAP3K13* |
| *SOX9* | *PLCE1* | *MAP3K13* | *EFNA5* |
| *ITGA6* | *PLAU* | *PRKAA2* | *VEGFA* |
| *POLD4* | *FGF19* | *PLCG2* | *POLB* |
| *LAMC2* | *CAPN2* | *DHRS3* | *TLR2* |

**Mouse Studies**

**1. Study design**

We examined drug effectiveness using a human CRC xenograft line grown in mice. The anti-tumor effect of drugs administered as single agents or in combination (Tables S2 & S3) was evaluated by assessing toxicity and tumor growth inhibition. Drug responses using PDX mice were evaluated at Champions Oncology (Hackensack NJ). These PDX models are fully genotyped and well-characterized in terms of PDX response to single agents. A suitable PDX line (CTG-2359) was selected for testing based on the *CTNNB1*-mutant genotype of the pdx CRC model.

The CTG-2359 human PDX line was derived from a colon cancer liver metastasis (stage IV) that occurred in a 59 year old Caucasian male. Tumor grade = undifferentiated. No prior systemic or radiation treatment. The tumor genotype is as follows: *CTNNB1* mutation (pSer45Pro), APC (missense variant - ENST00000457016.1:c.5209A&gt;C) &amp; deletion (3_prime_UTR_variant), Microsatellite instable (MST score 83.69), *KRAS* – pending, *TP53* – deletion (downstream variant modifier), *TGFRB* – deletion (high impact), *PIK3A* – single nucleotide variant (moderate impact).

Oral preparations were formulated by Champions Oncology staff. Starting doses for study agents were: Sulindac 171 mg/kg/day, 9-cis retinoic acid 3 different doses mg/kg/d, and Liarozole 40 ppm in a base diet (4 IU/g of vitamin A).

Animals were weighed and tumor dimensions measured twice weekly. The study endpoint was when the mean tumor volume of the control group (uncensored) reaches 1500mm^3^. If this occurred before Day 28, treatment groups and individual mice may be dosed and measured up to Day 28. If the mean tumor volume of the control group (uncensored) does not reach 1500mm^3^ by Day 28, then the endpoint for all animals were the day when the mean tumor volume of the control group (uncensored) reaches 1500mm^3^ up to a maximum of Day 60.

| **Table S5. Testing toxicity of drugs administered as single agents or in combination.** | | | | | |
| --- | --- | --- | --- | --- | --- |
| **Group** | **Agent** | **Dose Level** | **Dose Volume** | **ROA/Schedule** | **Mice** |
| 1 | Placebo | 0 mg/kg | 0 mL/kg | PO/ | 3 |
| 2 | Sulindac | 171 mg/kg | 0 mL | Medicated Chow/ | 3 |
| 3 | Liarozole | 40 mg/kg | 0 mL | Medicated Chow/ | 3 |
| 4 | 9-cis RA | 5 mg/kg | 10 mL/kg | PO/ | 3 |
| 5 | 9-cis RA | 10 mg/kg | 10 mL/kg | PO/ | 3 |
| 6 | 9-cis RA | 30 mg/kg | 10 mL/kg | PO/ | 3 |
| 7 | Sulindac | 171 mg/kg | 0 mL | Medicated Chow/ | 3 |
|  | 9-cis RA | 5 mg/kg | 10 mL/kg | PO/ |  |
| 8 | Sulindac | 171 mg/kg | 0 mL | Medicated Chow/ | 3 |
|  | 9-cis RA | 10 mg/kg | 10 mL/kg | PO/ |  |
| 9 | Sulindac | 171 mg/kg | 0 mL | Medicated Chow/ | 3 |
|  | 9-cis RA | 30 mg/kg | 10 mL/kg | PO/ |  |
| 10 | Liarozole | 40 mg/kg | 0 mL | Medicated Chow/ | 3 |
|  | 9-cis RA | 5 mg/kg | 10 mL/kg | PO/ |  |
| 11 | Liarozole | 40 mg/kg | 0 mL | Medicated Chow/ | 3 |
|  | 9-cis RA | 10 mg/kg | 10 mL/kg | PO/ |  |
| 12 | Liarozole | 40 mg/kg | 0 mL | Medicated Chow/ | 3 |
|  | 9-cis RA | 30 mg/kg | 10 mL/kg | PO/ |  |
| 13 | Sulindac | 171 mg/kg | 0 mL | Medicated Chow/ | 3 |
|  | Liarozole | 40 mg/kg | 0 mL | Medicated Chow/ |  |
| 14 | Sulindac | 171 mg/kg | 0 mL | Medicated Chow/ | 3 |
|  | Liarozole | 40 mg/kg | 0 mL | Medicated Chow/ |  |
|  | 9-cis RA | 5 mg/kg | 10 mL/kg | PO/ |  |
| 15 | Sulindac | 171 mg/kg | 0 mL | Medicated Chow/ | 3 |
|  | Liarozole | 40 mg/kg | 0 mL | Medicated Chow/ |  |
|  | 9-cis RA | 10 mg/kg | 10 mL/kg | PO/ |  |
| 16 | Sulindac | 171 mg/kg | 0 mL | Medicated Chow/ | 3 |
|  | Liarozole | 40 mg/kg | 0 mL | Medicated Chow/ |  |
|  | 9-cis RA | 30 mg/kg | 10 mL/kg | PO/ |  |

| **Table S6. Testing efficacy of drugs using PDX models.** | | | | | |
| --- | --- | --- | --- | --- | --- |
| **Group** | **Agent** | **Dose Level** | **Dose Volume** | **ROA/Schedule** | **Mice** |
| 1 | Placebo | 0 mg/kg | 0 mL/kg | PO/ | 8 |
| 2 | Sulindac | 171 mg/kg | 0 mL | Medicated Chow/ | 8 |
| 3 | Liarozole | 40 mg/kg | 0 mL | Medicated Chow/ | 8 |
| 4 | 9-cis RA | 30 mg/kg | 10 mL/kg | PO/ | 8 |
| 5 | Sulindac | 171 mg/kg | 0 mL | Medicated Chow/ | 8 |
|  | 9-cis RA | 30 mg/kg | 10 mL/kg | PO/ |  |
| 6 | Liarozole | 40 mg/kg | 0 mL | Medicated Chow/ | 8 |
|  | 9-cis RA | 30 mg/kg | 10 mL/kg | PO/ |  |
| 7 | Sulindac | 171 mg/kg | 0 mL | Medicated Chow/ | 8 |
|  | Liarozole | 40 mg/kg | 0 mL | Medicated Chow/ |  |
|  | 9-cis RA | 30 mg/kg | 10 mL/kg | PO/ |  |

**2. Sample size**

Eight immunocompromised mice (nunu Nude mice) for each treated PDX cohort were compared to 8 PDX control mice. Total # non-PDX mice for toxicity testing= 45. Total # PDX mice with human CRC xenografts = 56 mice per pdx model

**3. Inclusion and exclusion criteria**

Mice were excluded if they did not meet the following criteria: 1) Female gender:; 2) Age of 6-8 weeks at initiation of dosing; 3) At least 18 grams target weight at initiation of dosing. Naive animals were used for tolerability.

**4. Randomization**

Animals were randomized into treatment groups and dosing initiated once tumors reach a volume that falls within the range of 150-300mm^3^. The first day of the study was defined as the first day animals are dosed. Tumor measurements were taken on either Day 0 or Day 1.

**5. Blinding**

Only Champions Oncology staff were aware of the group allocation at the different stages of the experiment (during the allocation, the conduct of the experiment, the outcome assessment, and the data analysis).

**6. Outcome measures**

Acceptance Criteria: (1) Tumor growth inhibition (%TGI – see below) of PDXs by 50-100% in response to single drugs (Sulindac, Liarozole, 9-cis retinoic acid) versus placebo. (2) An increase in the anti-CRC effect of a multi-drug combination, compared to giving the drug alone, indicates synergistic or additive activity.

**7. Statistical methods**

The drug response for each PDX line was calculated based on tumor growth inhibition (%TGI) using the difference between the final and initial tumor volumes of the vehicle control as the baseline. The formula is %TGI = [1-(Treatment volume-final - Treatment volume-initial/Control volume-final – Control volume-initial)] *100. In selecting specific models to test, the level of power is set (0.80) to estimate how many mice were required for each group to have statistical significance. The power level calculation for the level of significance (alpha level or the false positive rate) was set at 0.05, where power is calculated based on the probability of identifying an actual difference if one exists (based on false negative rates).

**8. Experimental animals**

Test System

Species: Mouse

Strain: Athymic Nude-Foxn1nu (Immune-compromised)

Source: Charles River: Wilmington, Massachusetts

Gender: Female

Age at initiation of dosing: 6-8 weeks.

Target weight at initiation of dosing: At least 18 grams

Acclimation period: 3 days

Husbandry

Cages: Individual HEPA ventilated cages

Bedding: Irradiated corncob (Teklad 7902, CS)

Enrichment: 100% virgin kraft nesting enrichment sheets (Innorichment™)

Food: Sterilized test rodent diet (Teklad 2019), 19% protein, 9% fat and 4% fiber

Base chow diet +- gastric gavage (corn, soybean, or sesame oil)

Water: ad libitum reverse osmosis, 2 ppm Cl2

Environmental Conditions Temperature: 68-74°F (20-23°C) Humidity: 30-70%

Light cycle: 14-10 hour light-dark cycle

**9. Experimental procedures**

Data Collection and Reporting All data collected in this study were managed electronically. Animal weight and tumor measurement data were collected and managed electronically using Champions Laboratory Information Management System (BioIT) and stored on a redundant server system. Raw data updates was submitted via email once weekly using Champions standard template. Approximately four weeks following completion of the final model in the study a draft final report including associated tables, graphs, and raw data was submitted electronically utilizing Champions standard template for sponsor review and comment. Following draft report approval by sponsor, a final signed report was issued electronically.

Agent Toxicity: Beginning Day 0, animals were observed daily and weighed twice weekly using a digital scale; data including individual and mean gram weights (Mean Weight ± SEM), mean percent weight change versus Day 0 (%vD0) was recorded for each group and %vD0 plotted at study completion. Single agent or combination groups reporting a mean %vD0 >20% and/or >10% mortality were considered above the maximum tolerated dose (MTD) for that treatment on the evaluated regimen. Maximum mean %vD0 (weight nadir) for each treatment group was reported at study completion.

Tumor Growth Inhibition - Beginning Day 0, tumor dimensions were measured twice weekly by digital caliper and data including individual and mean estimated tumor volumes (Mean TV ± SEM) recorded for each group; tumor volume is calculated using the formula (1): TV= width2 x length x 0.52 . At study completion, percent tumor growth inhibition (%TGI) values was calculated and reported for each treatment group (T) versus control (C) using initial (i) and final (f) tumor measurements by the formula (2): %TGI = 1 - (Tf-Ti) / (Cf-Ci)*100. Individual mice reporting a tumor volume less than or equal to 30% of the Day 0 measurement for two consecutive measurements were considered partial responders (PR). Individual mice lacking palpable tumors (0.00 mm³ for two consecutive measurements) were classified as complete responders (CR); a CR that persists until study completion was considered a tumor-free survivor (TFS). Tumor doubling time (DT) was determined for the vehicle treated groups using the formula DT = (Df – Di) * log2 / (logTVf – logTVi) where D = Day and TV = Tumor Volume.

**10. Results**

**Tolerability Study**

Drug toxicity was initially evaluated for tolerability (Figure S3) based on body weight measurements. Body weights remained mostly stable with no weight loss greater than 10%. The triple combination treatment group maintained slightly lower but stable body weights, supporting an acceptable treatment regimen.

**Figure S5** Body weights for CTG-0000 A tolerability. Control mice tolerate both single and combination treatments. Naive animals were used for tolerability. Body weight measurements were taken over a 25-day tolerability study of CTG-0000 A. Multiple experimental treatments were monitored daily for changes in body mass. The y-axis represents body weight in grams, while the X-axis shows the progression of days (0-25). Different colored lines indicate various treatment conditions, including control and combination treatments. Body weights remained mostly stable, ranging from 23 to 27 grams across most groups, with one treatment group (orange line-Sulindac, 9-cis RA, and Liarozole high) showing a slight increase in body weight after day 15.

**Figure S6**. Tumor growth rate. Log10-transformed tumor volume (TV) measurements were taken over 28 days for efficacy. The y-axis represents the log10 of tumor volume, while the x-axis shows the days following treatment initiation (Panel **C**). The results show the linear growth patterns on a logarithmic scale, indicating exponential tumor growth. From approximately log10(TV) =2.5 at day 0, tumor volumes increased steadily to log10(TV) ≈ 3.3 by day 28. Error bars represent ± the SEM. The nearly parallel lines suggest a constant growth rate of placebo cases across treatment groups, with slight variations in the final tumor volumes. Measurements were taken at 5-day intervals throughout the study period.

**Efficacy Study**

The efficacy study evaluated treatment outcomes by monitoring the mice's body weight (Figure 8A) and measuring the tumor volume (Figure 8B). Raw tumor volume measurements indicated continuous growth across all treatment groups, with final volumes ranging from approximately 1000-2000 mm³ by day 25, while the triple combination treatment (Sulindac + Liarozole + 9-cis RA) demonstrated the lowest absolute tumor volumes throughout the study period. Log10-transformed data revealed linear growth patterns, suggesting that tumor growth followed exponential kinetics across all groups (Figure 8C). Regarding tolerability, absolute body weight measurements remained stable (22-28g range), indicating general treatment tolerability, and percent change analyses showed positive weight gain (3-13%) across most groups, implying minimal systemic toxicity. The triple combination treatment group maintained slightly lower, but stable body weights, supporting an acceptable treatment regimen. Notably, the correlation between treatment efficacy and tolerability showed that while the triple combination treatment provided the best response, it also maintained an acceptable therapeutic window. Single-agent treatments (Sulindac, Liarozole, 9-cis RA) resulted in higher tumor volumes, but demonstrated good tolerability with positive weight gain trends. The consistency of log-transformed growth curves suggested that while treatments influenced absolute tumor volume, they do not fully arrest tumor growth. In conclusion, the study indicates that the triple combination treatment therapy is the most promising treatment option, balancing efficacy and tolerability, with all treatments being generally well-tolerated based on stable or increasing body weights.

**References**

36. Zhang T, Fields JZ, Ehrlich SM, Boman BM. The Chemopreventive Agent Sulindac Attenuates Expression of the Antiapoptotic Protein Survivin in Colorectal Carcinoma Cells. J Pharmacol Exp Ther. 2004;308(2):434-437. doi:10.1124/jpet.103.059378

37. Rice PL, Kelloff J, Sullivan H, et al. Sulindac metabolites induce caspase- and proteasome-dependent degradation of beta-catenin protein in human colon cancer cells. Mol Cancer Ther. 2003;2(9):885-892. <http://www.ncbi.nlm.nih.gov/pubmed/14555707>

38. McEntee MF, Chiu CH, Whelan J. Relationship of beta-catenin and Bcl-2 expression to sulindac-induced regression of intestinal tumors in Min mice. Carcinogenesis. 1999;20(4):635-640. doi:10.1093/carcin/20.4.635

39. Mahmoud NN, Boolbol SK, Bilinski RT, Martucci C, Chadburn A, Bertagnolli MM. Apc gene mutation is associated with a dominant-negative effect upon intestinal cell migration. Cancer Res. 1997;57(22):5045-5050. http://www.ncbi.nlm.nih.gov/pubmed/9371501

40. Brown WA, Skinner SA, Vogiagis D, O’Brien PE. Inhibition of beta-catenin translocation in rodent colorectal tumors: a novel explanation for the protective effect of nonsteroidal antiinflammatory drugs in colorectal cancer. Dig Dis Sci. 2001;46(11):2314-2321. doi:10.1023/a:1012326525692

43. Marx J. Cancer research. Anti-inflammatories inhibit cancer growth--but how? Science. 2001;291(5504):581-582. doi:10.1126/science.291.5504.581

47. Rao C V, Rivenson A, Simi B, et al. Chemoprevention of colon carcinogenesis by Sulindac, a nonsteroidal anti-inflammatory agent. Cancer Res. 1995;55(7):1464-1472. http://www.ncbi.nlm.nih.gov/pubmed/7882354

48 Thompson WJ, Piazza GA, Li H, Liu L, Fetter J, Zhu B, Sperl G, Ahnen D, Pamukcu R. Exisulind induction of apoptosis involves guanosine 3',5'-cyclic monophosphate phosphodiesterase inhibition, protein kinase G activation, and attenuated beta-catenin. Cancer Res. 2000 Jul 1;60(13):3338-42.

49. Saini MK, Sanyal SN. Piroxicam and c-phycocyanin prevent colon carcinogenesis by inhibition of membrane fluidity and canonical Wnt/β-catenin signaling while up-regulating ligand dependent transcription factor PPARγ. Biomed Pharmacother. 2014 Jun;68(5):537-50. doi: 10.1016/j.biopha.2014.03.007.

50. Ritland SR, Gendler SJ. Chemoprevention of intestinal adenomas in the ApcMin mouse by Piroxicam: kinetics, strain effects and resistance to chemosuppression. Carcinogenesis. 1999 Jan;20(1):51-8. doi: 10.1093/carcin/20.1.51.

51. Li H, Kramer PM, Lubet RA, Steele VE, Kelloff GJ, Pereira MA. Termination of Piroxicam treatment and the occurrence of azoxymethane-induced colon cancer in rats. Cancer Lett. 1999;147(1-2):187-193. doi:10.1016/s0304-3835(99)00296-7

52. Rao C V, Tokumo K, Rigotty J, Zang E, Kelloff G, Reddy BS. Chemoprevention of colon carcinogenesis by dietary administration of Piroxicam, alpha-difluoromethylornithine, 16 alpha-fluoro-5-androsten-17-one, and ellagic acid individually and in combination. Cancer Res. 1991;51(17):4528-4534. http://www.ncbi.nlm.nih.gov/pubmed/1831401

61. Boon, E.M.J.; Keller, J.J.; Wormhoudt, T.A.M.; Giardiello, F.M.; Offerhaus, G.J.A.; van der Neut, R.; Pals, S.T. Sulindac targets nuclear _-catenin accumulation and Wnt signaling in adenomas of patients with familial adenomatous polyposis and in human colorectal cancer cell lines. Br. J. Cancer **2004**, 90, 224–229.

62. Han, A.; Song, Z.; Tong, C.; Hu, D.; Bi, X.; Augenlicht, L.H.; Yang, W. Sulindac suppresses _-catenin expression in human cancer cells. Eur. J. Pharmacol. **2008**, 583, 26–31.

63. Lu, H.N.; Gary, B.D.; Keeton, A.B.; Lu, W.; Li, Y.; Piazza, G.A. Inhibition of PDE5 by sulindac sulfide selectively induces apoptosis and attenuates oncogenic Wnt/beta-catenin-mediated transcription in human breast tumor cells. Cancer Prev. Res. **2011**, 4, 1275–1284.

64. Bhatlekar S, Addya S, Salunek M, Orr C, Surrey S, McKenzie S, Fields J, Boman BM. Identification of a developmental gene expression signature, including HOX genes, for the normal human colonic crypt stem cell niche – Overexpression of the signature parallels stem cell overpopulation during colon tumorigenesis Stem Cells Dev 23:167-79, 2014.

65. Stein, U.; Arlt, F.; Smith, J.; Sack, U.; Herrmann, P.; Walther, W.; Lemm, M.; Fichtner, I.; Shoemaker, R.H.; Schlag, P.M. Intervening in _-catenin signaling by sulindac inhibits s100a4-dependent colon cancer metastasis. Neoplasia **2011**, 13, 131–144.

66. Lu W, Tinsley HN, Keeton A, Qu Z, Piazza GA, Li Y. Suppression of Wnt/beta-catenin signaling inhibits prostate cancer cell proliferation. Eur J Pharmacol. 2009 Jan 5;602(1):8-14. doi: 10.1016/j.ejphar.2008.10.053.

67. Steinert, G.; Oancea, C.; Roos, J.; Hagemeyer, H.; Maier, T.; Ruthardt, M.; Puccetti, E. Sulindac sulfide reverses aberrant self-renewal of progenitor cells induced by the AML-associated fusion proteins PML/RAR and PLZF/RARalpha. PLoS ONE **2011**, 6, e22540.

68. Bank, A.; Yu, J.; Zhang, L. NSAIDs downregulate Bcl-X(L) and dissociate BAX and Bcl-X(L) to induce apoptosis in colon cancer cells. Nutr. Cancer 2008, 60 (Suppl. 1), 98–103.

69. Lee, H.-C.; Park, I.-C.; Park, M.-J.; An, S.; Woo, S.-H.; Jin, H.-O.; Chung, H.Y.; Lee, S.-J.; Gwak, H.-S.; Hong, Y.-J.; et al. Sulindac and its metabolites inhibit invasion of glioblastoma cells via down-regulation of Akt/PKB and MMP-2. J. Cell. Biochem. **2005**, 94, 597–610.

70. Paw, I.; Carpenter, R.C.; Watabe, K.; Debinski, W.; Lo, H.-W. Mechanisms regulating glioma invasion. Cancer Lett. **2015**, 362, 1–7.

71. Lee, H.J.;Wang, N.X.; Shi, D.L.; Zheng, J.J. Sulindac inhibits canonical Wnt signaling by blocking the PDZ domain of the protein Dishevelled. Angew. Chem. **2009**, 48, 6448–6452.

72. Tinsley, H.N.; Gary, B.D.; Keeton, A.B.; Lu, W.; Li, Y.; Piazza, G.A. Inhibition of PDE5 by sulindac sulfide selectively induces apoptosis and attenuates oncogenic Wnt/_-catenin-mediated transcription in human breast tumor cells. Cancer Prev. Res. **2011**, 4, 1275–1284.

73. Li, N.; Xi, Y.; Tinsley, H.N.; Gurpinar, E.; Gary, B.D.; Zhu, B.; Li, Y.; Chen, X.; Keeton, A.B.; Abadi, A.H.; et al. Sulindac selectively inhibits colon tumor cell growth by activating the cGMP/PKG pathway to suppress Wnt/_-catenin signaling. Mol. Cancer Ther. **2013**, 12, 1848–1859.
